# LC-MS/MS profiling of *Tipuana tipu* flower, HPLC-DAD quantification of its bioactive components, and interrelationships with antioxidant, and anti-inflammatory activity: in vitro and in silico approaches

**DOI:** 10.1186/s12906-024-04467-5

**Published:** 2024-04-26

**Authors:** Rana M. Ibrahim, Passent M. Abdel-Baki, Ahmed A. El-Rashedy, Nariman E. Mahdy

**Affiliations:** 1 Pharmacognosy Department, Faculty of Pharmacy, Cairo University, Kasr-El-Ainy Street, Cairo, 11562 Egypt; 2 Natural and Microbial Products Department, National Research Center (NRC), Dokki, Giza, 12622 Egypt

**Keywords:** *Tipuana tipu*, Antioxidant, Anti-inflammatory, LC-QTOF-MS/MS, Flavonoids, Molecular docking.

## Abstract

**Background:**

Fabaceae plays a crucial role in African traditional medicine as a source of large number of important folk medication, agriculture and food plants. In a search of potential antioxidant and anti-inflammatory candidates derived from locally cultivated plants, the flowers of *Tipuana tipu* (Benth.) Lillo growing in Egypt were subjected to extensive biological and phytochemical studies. The impact of the extraction technique on the estimated biological activities was investigated.

**Methods:**

The flowers were extracted using different solvents (aqueous, methanol, water/methanol (1:1), methanol/methylene chloride (1:1), and methylene chloride). The different extracts were subjected to antioxidant (DPPH, ABTS, and FRAP) and anti-inflammatory (COX-2 and 5-LOX) assays. The methanol extract was assessed for its inhibitory activity against iNOS, NO production, and pro-inflammatory cytokines (NF-KB, TNF-R_2_, TNF-*α*, IL-1*β*, and IL-6) in LPS-activated RAW 264.7 macrophages. The composition-activity relationship of the active methanol extract was further investigated using a comprehensive LC–QTOF-MS/MS analysis. The major identified phenolic compounds were further quantified using HPLC-DAD technique. The affinity of representative compounds to iNOS, COX-2, and 5-LOX target active sites was investigated using molecular docking and molecular dynamics simulations.

**Results:**

The methanol extract exhibited the highest radical scavenging capacity and enzyme inhibitory activities against COX-2 and 5-LOX enzymes with IC_50_ values of 10.6 ± 0.4 and 14.4 ± 1.0 µg/mL, respectively. It also inhibited iNOS enzyme activity, suppressed NO production, and decreased the secretion of pro-inflammatory cytokines. In total, 62 compounds were identified in the extract including flavonoids, coumarins, organic, phenolic, and fatty acids. Among them 18 phenolic compounds were quantified by HPLC-DAD. The highest docking scores were achieved by kaempferol-3-glucoside and orientin. Additionally, molecular dynamics simulations supported the docking findings.

**Conclusion:**

The flower could be considered a potentially valuable component in herbal medicines owing to its unique composition and promising bioactivities. These findings encourage increased propagation of *T. tipu* or even tissue culturing of its flowers for bioprospecting of novel anti-inflammatory drugs. Such applications could be adopted as future approaches that benefit the biomedical field.

**Supplementary Information:**

The online version contains supplementary material available at 10.1186/s12906-024-04467-5.

## Background

Family Fabaceae is the third largest flowering plants family, exceeded only by the Orchidaceae and Compositae [1]. It comprises about 730 genera and more than 19,400 species. Plants of this family grow in a variety of habitats and climates throughout the world. Many are important food and agricultural plants, including *Glycyrrhiza glabra* (licorice), *Glycine max* (soybean), *Medicago sativa* (alfalfa), *Phaseolus* (beans) and *Pisum sativum* (pea) [2]. The Genus *Tipuana* (Benth.) Benth. (Subfamily Papilionoideae) is represented by *Tipuana tipu* (Benth.) Lillo species, a South African tree. Also, it is commonly grown in several other countries, such as Egypt, Australia, Argentina, Uruguay, Paraguay, Brazil, and Bolivia [3, 4]. It is an ornamental tree and is also used as a supplementary food to ruminants (e.g., sheep), mostly the leaves which showed high nutritive value [5]. *T. tipu* was recommended for wound healing, hemorrhoids, gastrointestinal tract disorders, and abdominal and rheumatic pains. It has anti-inflammatory and free-radical scavenging activities, in addition it is used as an astringent for its high tannin content [6, 7]. Ethyl acetate and methylene chloride extracts of the aerial parts of *T. tipu* were proved to be efficient in inhibiting *Listeria monocytogenes* and *Staphylococcus aureus* growth. Moreover, leaf extracts showed promising nephroprotective and antimalarial activities [8]. Few phytochemical studies were performed on the leaf and the bark of *T. tipu* isolating formononetin, *β*-sitosterol, *β*-sitosterol glucoside, lupeol, *α*-amyrin, 1-nonadecanol, alpinumisoflavone, protocatechualdehyde, protocatechuic acid, and stearic acid [3, 5, 9, 10]. A kaempferol glycoside with marked anti-inflammatory activity, kaempferol 3-O-*α*-L-rhamnopyranosyl-(1→6)-O-[*β*-D-glucopyranosyl-(1→2)-4-O-acetyl-*α*-L-rhamnopyranosyl-(1→2)]-*β*-D-galactopyranoside, has been isolated from *T. tipu* (Benth.) Lillo leaves growing in Egypt, with another three flavonol glycosides, kaempferol 3-O-rutinoside, rutin, and kaempferol-3-O-[*α*-L-rhamnopyranosyl-(1→6)]-[*α*-L-rhamnopyranosyl-(1→2]-*β*-glucopyranoside [11].

Despite all progress in synthetic biotechnology and chemistry, herbs are still indispensable sources of medicinal preparations and considered as an important part of healthcare throughout the world [12]. In addition, plant-based medications have emerged as a promising source for developing new pharmaceuticals and functional items due to the side effects of synthetics [13, 14], along with their higher safety margin and lower cost [15]. Surveying the current literature, all studies mainly focused on the leaves of *T. tipu* (Benth.) Lillo. Although flowers are considered as rich sources for valuable constituents depending on our phytochemical investigations, few and incomplete studies were traced concerning their contents and antioxidant and anti-inflammatory bioactivities [16]. Accordingly, different solvent extracts of the flowers (aqueous, methanolic, water/methanol (1:1), methanol/methylene chloride (1:1), and methylene chloride) were tested for their antioxidant capacities using different methods, such as DPPH (2,2-diphenyl-1-picrylhydrazyl), ABTS (2,2’-azino-bis(3-ethylbenzthiazoline-6-sulfonic acid)), and FRAP (ferric reducing antioxidant power). Furthermore, the in vitro anti-inflammatory (COX-2 and 5-LOX) effects of all extracts were evaluated. Then, the most active extract (methanolic extract) was evaluated for its inhibitory activity against the inducible nitric oxide synthase enzyme (iNOS), nitric oxide production (NO), and the secretion of the pro-inflammatory cytokines (NF-KB, TNF-R2, TNF-*α*, IL-1*β*, and IL-6) in LPS-activated RAW 264.7 macrophages. As well as it was of a great interest to provide a comprehensive metabolic profiling of *T. tipu* flowers using an integrated approach of LC-QTOF-MS/MS and HPLC-DAD analyses. Further, computational study using molecular docking simulations with iNOS, COX-2, and 5-LOX active sites was performed to establish for the first time the correlation between the elucidated chemical profiles and the anti-inflammatory effects of flowers. Thus, this study aims to provide a holistic understanding of the phytochemical composition of *T. tipu* flowers in relation to their biological potential, as the literature lacks such detailed information. Such an approach is required for future breeding programs alongside quality control purposes that offer chemical-based evidence regarding *T. tipu* biological potential and health benefits.

## Materials and methods

The detailed procedures are described in the Supplementary online resource.

### Plant material

Flowers of *Tipuana tipu* (Benth.) Lillo were collected during Spring season (March 2022) from the Zoo (Giza, Egypt). The taxonomic identity of the plant material was kindly authenticated and verified by Mrs. Teresa Labib, Head of the Taxonomists at Orman Botanic Garden, Egypt. This study complies with local, national, and international guidelines, and no specific consent was required for the collection of the plant material.

### Preparation of different flower extracts

The flowers (1.5 kg) were air-dried, grinded, and extracted separately in triplicates (500 g each) with water, methanol, water/methanol (1:1), methanol/methylene chloride (1:1), and methylene chloride (twice, 3 L each) by cold maceration. The extracts were then dried under vacuum in a rotary evaporator at 50 ºC and the residues were kept separately in tight containers until analysis. Finally, the dried residues (30 mg each) were dissolved separately in DMSO for the biological assays and the dried methanolic residue (10 mg) was dissolved in methanol for LC-MS analysis. Three biological replicates were prepared for each solvent type and extracted in parallel under the same conditions (*n* = 3).

### LC/QTOF-MS/MS analysis

*T. tipu* flowers methanolic extract was subjected to *metabolic* analysis using a 6530 Q-TOF LC/MS (Agilent Technologies, Santa Clara, CA, USA) outfitted with an autosampler (G7129A), a quaternary pump (G7104C). The extract was separated on a Zorbax RP-18 column (150 mm × 3 mm, dp = 2.7 μm, Agilent Technologies). The flow rate was 0.2 mL/min, and the injection volume was 2 µL. Water (A) and acetonitrile (B) with formic acid (0.1%) in each utilized as the solvents and applied using a gradient elution as follows 5% B linearly increased to 90% B within 30 min, then remained isocratic at 100% B for 2 min before linearly decreasing back to 5% B for the following 5 min. Mass spectra were obtained using ESI in negative ionization mode using the conditions previously described [17]. Briefly, the Q-TOF mass spectrometer was optimized in negative ESI mode with the following parameters: capillary voltage 3500 V, drying gas temperature and flow rate 350 °C and 8 L/min, respectively, nebulizer gas 30 psi, fragmentor voltage 150 V, skimmer voltage 60 V, and OCT 1 RF Vpp voltage 300 V. The auto-MS/MS mode was used in the m/z range of 50-2000, with an MS scan rate of 10 spectra/s and an MS/MS scan rate of 6 spectra/s. The precursor selection criteria were as follows: maximum precursor per cycle 9, absolute threshold 200 counts, relative threshold 0.01%, purity stringency 100%, purity cutoff 30%, precursors sorted by abundance only, and isolation window ∼ 1.3 m/z (narrow). Mass Hunter software (ver. B.06, Agilent Technologies, Inc. 2012) was used for instrument control, data acquisition, and processing of the MS/MS spectra. Metabolites were characterized by their retention times (min), exact molecular ion mass (M-H)^–^, MS/MS fragment ions relative to accessible databases, such as the Human Metabolome Database (http://www.hmdb.ca/) and lipidMaps (https://www.lipidmaps.org/).

## Results and discussion

### Antioxidant and inhibitory activities against COX-2 and 5-LOX of different flower extracts

**Fig. 1:**
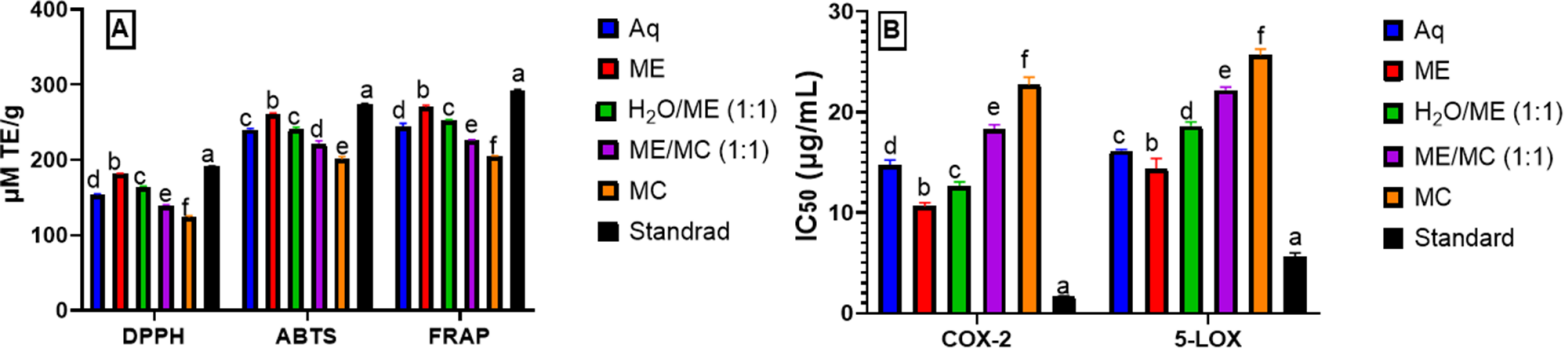
(A) Antioxidant (DPPH ABTS and FRAP methods) and (B) inhibitory activities of *T. tipu* flowers aqueous, methanolic, water/methanol (1:1), methanol/methylene chloride (1:1) and methylene chloride extracts against COX-2 and 5-LOX

The pharmaceutical industry is transitioning towards nature-derived antioxidants because of the adverse effects associated with synthetic drugs [18]. The capability of various natural constituents to reduce inflammation is supposed to be from the following: firstly, acting as antioxidants; then, interfering with the signaling of free radical species; finally, decreasing the pro-inflammatory signaling transductions [19]. Therefore, the antioxidant (ABTS, FRAP, and DPPH methods), as well as inhibitory activities against COX-2 and 5-LOX of aqueous, methanol, 50% aqueous methanol, methylene chloride, and 50% methylene chloride/methanol extracts of the flowers were assessed using in vitro assays (Fig. 1), in an attempt to understand the ability of these extracts to develop effective intervention for inflammatory disease prevention strategies. Herein, the methanolic extract exhibited the highest antioxidant potential equivalent to 181.5 ± 0.8 µM TE/g (DPPH), 261.1 ± 1.1 µM TE/g (ABTS), and 270.3 ± 2.5 µM TE/g (FRAP), in comparison to ascorbic acid (standard drug). Relative to the other extracts, the methanol extract showed the highest inhibition against COX-2 (IC_50_ 10.6 ± 0.4 µg/mL) compared to Celecoxib (IC_50_ 1.70 ± 0.0 µg/mL), and the highest 5-LOX inhibitory potential (IC_50_ 14.4 ± 1.0 µg/mL) compared to Zileuton as a standard (IC_50_ 5.65 ± 0.4 µg/mL).

Aq: aqueous extract; H_2_O/ME: water/methanol (1:1); MC: methylene chloride extract; ME: methanolic extract; ME/MC: methanol/methylene chloride (1:1) extract. Data are represented as mean ± standard deviation of three replicates. Different letters on the bar imply significant differences at *P* < 0.0001 with Tukey’s test.

Considering the fact that the methanol extract exhibited the highest radical scavenging potential and enzyme inhibitory activities, it is important to further ascertain its anti-inflammatory behavior. Therefore, its inhibitory effects against iNOS enzyme, NO production, and pro-inflammatory cytokines secretion (TNF-*α*, IL-1*β*, IL-6, NF-KB, and TNF-R2) in LPS-activated RAW 264.7 macrophages were evaluated.

### Cell viability on RAW264.7 macrophages

In MTT assay, *T. tipu* flowers methanolic extract (ME) did not show cytotoxic activity (up to 500 µg/mL) (Fig. S1) when assayed on RAW264.7 macrophages (after 24 h incubation), indicating ideal safety profile of the methanolic extract so it can be used in alleviating painful disease symptoms and improving human health.

### Inhibitory activity of methanolic extract (ME) of T. tipu flowers against iNOS enzyme activity, NO production, and secretion of pro-inflammatory cytokines in LPS stimulated RAW 264.7 macrophages

The excess production of inflammatory mediators in many ailments, like asthma, arthritis, vascular disease, obesity, and dermatitis has become one of the first global morbidity causes [20]. Inflammatory mediators, such as nitric oxide (NO) and pro-inflammatory cytokines [tumor necrosis factor-*α* (TNF-*α*), interleukin (IL)- IL-6, and IL-1*β*] are produced by lipopolysaccharide (LPS, a gram-negative bacteria) through activation of surface receptors, such as tumor necrosis factor receptors (TNF-R2), several protein kinases (MAPKs; extracellular signal-regulated kinase [ERK] and c-Jun N-terminal kinase [JNK]), and transcriptional factors as nuclear factor-κB (NF-κB) in macrophages [21]. In addition, LPS-stimulated macrophages exhibit up-regulation of iNOS expression through the generation of inflammatory cytokines. Therefore, inhibition of iNOS, inflammatory mediators and proinflammatory cytokines in LPS-stimulated macrophages can provide an effective approach for prevention of inflammatory disorders [22].

**Fig. 2:**
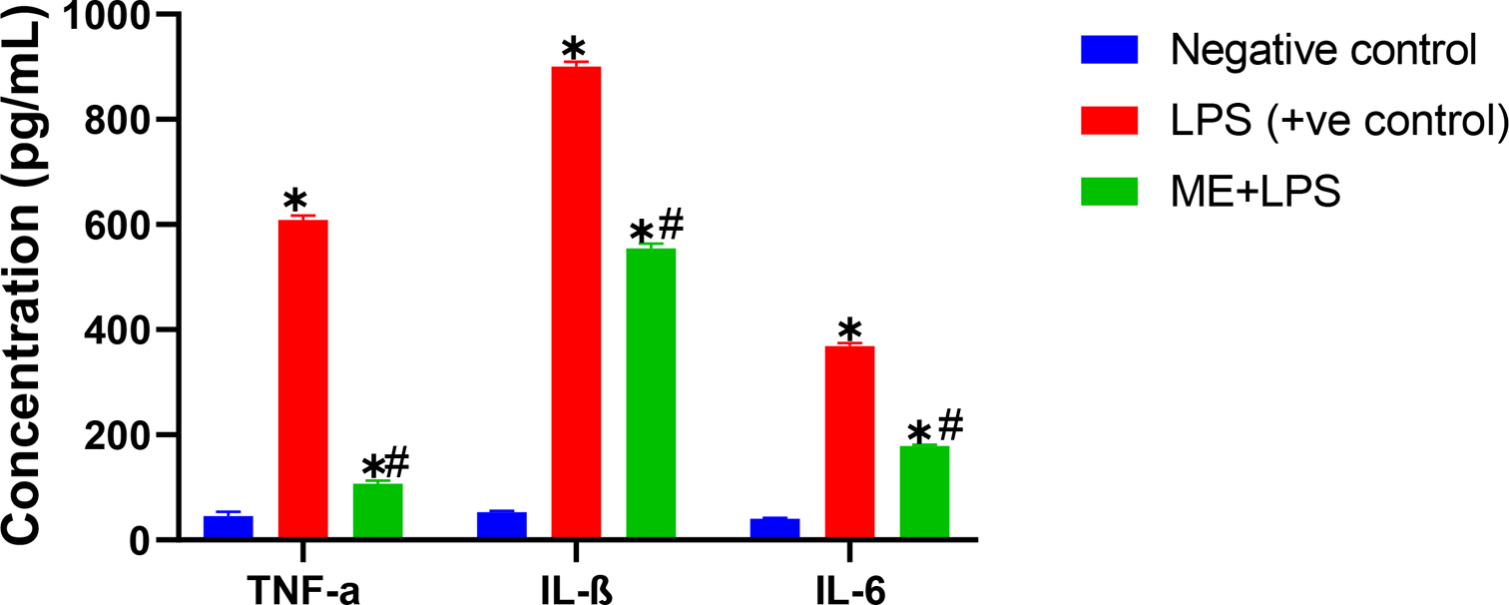
The inhibitory effect of methanolic extract (ME) of *T. tipu* flowers on pro-inflammatory cytokines (TNF-*α*, IL-*β*, and IL-6) production in LPS stimulated RAW 264.7 macrophages. ME: methanolic extract. * Significant from negative control at *P* < 0.0001. # Significant from positive control at *P* < 0.0001 with Tukey’s test

The ME of *T. tipu* flowers inhibited iNOS in LPS-induced RAW264.7 macrophages, with IC_50_ value of 11.1 ± 1.0 µM relative to parthenolide as a standard drug (IC_50_ 2.2 ± 0.0 µM). The results also indicated that the ME of the flowers is an effective inhibitor of LPS-induced NO production in RAW 264.7 cells and decreased the release of TNF-*α*, IL-*β*, IL-6, NF-KB, and TNF-R2, compared to standard drugs (Table 1; Fig. 2). These findings imply that ME of the flowers might be utilized as a natural anti-inflammatory resource. This prompted the use of LC-MS metabolic profiling of the ME to enable the preliminary identification of key components that may contribute synergistically to the anti-inflammatory and antioxidant effects, followed by their quantification and verification using computational analyses that could explain the structure-activity relationships.

**Table 1:** Inhibitory activity of *T. tipu* flowers methanolic extract (ME) against NO, NF-KB, and TNF-R2 production in LPS stimulated RAW 264.7 macrophages

Tested sample	Inhibition (IC_50_)		TNF-R2 (ng/mL)
	NO (µg/mL)	NF-KB (pg/mL)	
Methanol extract	8.51 ± 0.21*	9.33 ± 0.03*	7.63 ± 0.03 *
Reference drug	^A^3.5 ± 0.3	^C^7.53 ± 0.03	^B^6.81 ± 0.21

Values are represented as mean ± SD; ^A^: parthenolide; ^B^: Certolizumab; ^C^: curcumin; NF-KB: nuclear factorkappa B; NO: nitric oxide; TNF-R2: tumor necrosis factor-receptor 2; *Significantly different (*P* < 0.05) compared to reference drug using sample t-test.

### Metabolite profiling

The deficit of reports concerning the chemical profiles of the flowers under investigation motivated the performance of this study to investigate the chemical composition of *T. tipu* flowers through a non-targeted metabolite profiling of the prepared ME for detecting and identifying large numbers of metabolites using ultra performance liquid chromatography (UPLC) coupled with mass spectrometry (MS). Sixty-two compounds have been tentatively identified in the methanolic extract of *T. tipu* flowers after careful inspection of MS/MS data, comparison with on line database and reported literature [11, 23]. The compounds belonged to various classes encompassing: 2 sugars, 6 amino acids, 6 organic acids, 14 phenolic acids, 2 coumarins, 12 flavonoids, 8 fatty acids, and 12 phospholipids. Details of the tentatively identified compounds, including retention times, *m/z* of the detected molecular ions, fragment ions, and molecular formulas, as well as putative identifications are tabulated in Table 2. The base peak chromatogram of the analyzed extract in the negative ionization mode is shown in Fig S2. (supplementary materials). The representative MS/MS spectra of selected compounds from each class are displayed in Figs. S3-11. Interestingly, the main identified compounds were phenolic acids and flavonoids (a total of 26), agreeing with the previous reports for *T. tipu leaves* [11, 24].

#### Identification of phenolic acids

Phenolic compounds contribute significantly to antioxidant activity [25]. Fourteen phenolic acids belonged to various classes have been identified from their exact masses and fragmentation patterns [23]. They were mainly benzoic and hydroxycinnamic acid derivatives and were eluted after organic acids in the chromatographic separation. In the MS/MS spectra of phenolic acids, the loss of CO_2_ group from the carboxylic acid moiety (-44 amu), loss of CO group (-28 amu), as well as the loss of water molecule (-18 amu) led to the formation of characteristic fragment ions [26]. Five free benzoic acids have been identified i.e., benzoic acid, hydroxy benzoic acid, methoxy benzoic acid, protocatechuic acid, and vanillic acid. Two benzoic acid glycosides, protocatechuic acid glucoside and vanillic acid glucoside, were also detected with molecular ions at m/z 315.0721 and 329.0876, respectively, and MS^2^ spectra due to loss of glucose moiety (-162 amu) and CO_2_ group. Seven hydroxycinnamic acids were identified caffeic acid glucoside, *p*-coumaric acid glucoside, chlorogenic acid, cinnamic acid, ferulic acid, coumaric acid pentoside, and dihydrocaffeic acid glucoside. Cinnamic and ferulic acids produced molecular ions (M-H)^–^ at *m/z* 147.0655 and 193.0494, respectively, and MS^2^ spectra due to removal of CO_2_ group from the carboxylic acid function (at *m/z* 103 and 149, respectively). Chlorogenic acid showed a molecular ion peak at *m/z* 353.0873 (C_16_H_17_O_9_^–^) and a base peak at *m/z* 191 corresponding to the deprotonated quinic acid. Two glycosides of coumaric acid have been identified with molecular ions at *m/z* 295.0456 (C_13_H_11_O_8_^–^) and 325.0914 (C_7_H_13_O_7_^–^). Both showed main fragment ions at *m/z* 163 and 119 of coumaric acid and its decarboxylated form. Thus, they were identified as coumaric acid pentoside (Fig. S3) and coumaric acid glucoside (Fig. S4), respectively. Caffeic acid glucoside and dihydrocaffeic acid glucoside have been identified from their molecular ions at *m/z* 341.0879 and 343.1021, respectively. In their MS/MS spectra, they produced fragment ions due to loss of the sugar moiety to give the free acids at *m/z* 179 and 181, respectively, besides other characteristic ions due to sequential losses of CO, CO_2_, and H_2_O groups. The methanol extract demonstrated high phenolic acids content suggesting its antioxidant potential. Additionally, phenolic acids were found to have anti-inflammatory and protective effects against many oxidative stress related diseases viz. cancers, diabetic and cardiovascular disorders [27, 28]. Because of their phenol moiety and resonance-stabilized structure, phenolic acids have antioxidant properties due to electron and H-atom donations and radical quenching mechanisms [27, 29, 30].

### Identification of coumarins

Esculetin and its glycoside esculin have been detected in the *T. tipu* extract for the first time. They displayed molecular ion peaks at *m/z* 177.0128 and 339.0724, respectively. Esculetin (Fig. S5) showed typical fragmentation pattern of coumarins including successive losses of CO_2_ (-44 amu) and CO (-28 amu) groups from the molecular ion to produce three main fragment ions at *m/z* 133 [(M-H)-44]^–^, 105 [(M-H)-44-28]^–^, and 89 [(M-H)-44*2]^–^ [31]. While esculin (Fig. S6) gave its base peak at *m/z* 177 due to the breakdown of the glycosidic linkage and removal of the glucose moiety (-162 amu). Several authors have pointed to the varied range of pharmacological effects of esculetin and esculin, including antioxidant, anti-inflammatory, anticancer, antidiabetic, and neuroprotective [32, 33]. In vivo, esculin is metabolized into esculetin through phase I reaction [32]. Currently, esculetin has been shown in a number of studies to prevent the formation of reactive oxygen species (ROS), decrease the reduction of antioxidant enzymes, and suppress the activation of mitochondria-induced apoptotic pathways, thereby offering protection of cells from oxidative stress-induced apoptosis. It could also inhibit the release of proinflammatory cytokines, including tumor necrosis factor (TNF)-*α*, interleukin (IL)-l*β*, IL-2, and interferon (IFN)-*γ*, leading to decrease the migration of inflammatory cells. Besides, esculetin inhibited the activation of nuclear factor kappa B (NF-κB), the expression of inducible nitric oxide synthase (iNOS), and the cyclooxygenase-2 protein by blocking the NF-κB pathway and lowering the generation of pro-inflammatory mediators such as nitric oxide (NO) and prostaglandin E2 [32, 34]. It is worth noting that coumarins, i.e., esculin and esculetin are first reported in *T. tipu* flowers.

### Identification of flavonoids

Flavonoids are the most abundant group of plant polyphenolic compounds; they play a vital role in plant physiology [35] and are commonly found as glycosides bound to one or more sugar moieties through C- and/or O-linkage. Increasing scientific evidence suggested that flavonoids could have antioxidant and anti-inflammatory potential by suppressing regulatory enzymes or transcription factors implicated in the control of inflammatory mediators [36]. They exhibited neuroprotective effects against different neurodegenerative diseases, including Alzheimer, Parkinson, multiple sclerosis, and others through inhibiting pro-inflammatory cytokines expression, inflammatory markers, and thus preventing neural damage [37]. Several anti-inflammatory flavonoids have been reported in *T. tipu* leaves [11, 24]. Interestingly, 12 flavonoids have been tentatively identified in the methanol extract of *T. tipu* flowers for the first time.

Nine flavonols were detected in the extract of the flowers (Table 2). The identification of these compounds was facilitated by the analysis of fragmentation patterns of their molecular ions and the observation of glycosidic residues [rhamnosyl (-146 amu) and glucosyl (-162 amu)] that were cleaved sequentially and generated the characteristic aglycone, then its fragments, and all were compared to the reported data [23, 38]. Among them, 4 compounds were identified as kaempferol glycosides and 2 were quercetin glycosides, along with the aglycone (quercetin). In addition, two isorhamnetin glycosides were identified. In details, the MS^2^ spectra of isorhamnetin 3-glucoside [*m/z* 477.1026, C_22_H_21_O_12_^–^], quercetin 3-glucoside [*m/z* 463.0837, C_21_H_19_O_12_^–^], and kaempferol 3-glucoside [*m/z* 447.0903, C_21_H_19_O_11_^–^] showed removal of the attached glucose unit [M-H-162]^–^ and revealed base peaks at *m/z* 315 (isorhamnetin), 300 (quercetin) and 285 (kaempferol), respectively, confirming O-glycosylated flavonoid. Moreover, the rutinoside glycoside of kaempferol and quercetin have been identified depending on the fragment ions resulted from the consecutive loss of rhamnose and glucose units leading to the base peak at *m/z* 285 and 300, respectively, corresponding to the free aglycones. It is worth noting that the forementioned flavonol glycosides have been previously isolated from *T. tipu* leaves and were detected here in the flowers. Kaempferol 3,7-dirhamnoside (kaempferitrin) was identified depending on its molecular ion peak at *m/z* 577.1569 corresponding to C_27_H_29_O_14_^–^ and its fragmentation behavior. The MS/MS spectrum showed 2 main fragment ions at *m/z* 431 and 285 (Fig. S7) produced from successive loss of one and two rhamnose units, respectively. Another two flavonol glycoside, narcissin and kaempferol 3-O-rhamnosyl rutinoside were identified depending on their accurate masses, fragmentation patterns, and literature [23, 24].

Two compounds were mono-C-glycosyl flavones producing MS fragmentation patterns characteristic of C-glycosides flavonoids, including cross ring cleavage of the glucose moiety that produced fragmentation pattern of [(M-H)-120]^–^ and [(M-H)-90]^–^ [23]. Orientin and vitexin displayed molecular ions at *m/z* 447.0907 and 431.0964, respectively. In their MS^2^ spectra, both exhibited fragmentation pattern of [(M-H)-90]^–^, [(M-H)-120]^–^, yielding ions at *m/z* 357, 327 for orientin, respectively, and *m/z* 341, 311 for vitexin (Fig. S8), respectively. The lack of a loss of [(M-H)-H_2_O]^–^, confirming the sugar substitution in position 8 [38]. As authors are aware, this is the first report of C-glycosylated flavonoids in *T. tipu* plant.

### Identification of organic and fatty acids

Six organic acids were identified in the methanol extract (Table 2). They were coeluted early with amino acids and sugars and observed in the first half of the chromatogram (Fig. S2). Their fragmentation behavior showed remarkable losses of CO_2_ (-44 amu) and H_2_O (-18 amu) (Table 2). For instance, succinic acid (Fig. S9) showed (M-H)^–^ at *m/z* 117.0191 and product ions at *m/z* 99 [(M-H)-18]^–^ and 73 [(M-H)-44]^–^. While fatty acids were eluted later and appeared in the last half of the chromatogram (Fig. S2). The LC/MS analysis of the extract revealed many unsaturated fatty acids e.g., linoleic, linolenic acids, hydroxylated fatty acids e.g., trihydroxy octadecatrienoic acid and hydroxy eicosanoic acid, as well as saturated fatty acids e.g., palmitic acid. Figure S10 showed the fragmentation of palmitic acid as a representative of the identified fatty acids. It showed the main ions at *m/z* 255 [M-H]^–^, 211 [M-H-CO_2_]^–^, and 196 [M-H-CH_3_]^–^.

It is well documented that organic acids play an important role as antimicrobial, anti-inflammatory, and antioxidant agents. Furthermore, organic acids are crucial to biological processes because they are intermediate or end products in numerous essential pathways in the metabolism of plants and animals, playing a key role in the citric acid cycle or Krebs cycle [39]. Whereas fatty acids provide energy to the human body and serve as structural material for cell membranes and organ padding. They play a role in vitamin A and D absorption, blood clotting, and immune response. Some of them are prostaglandins and leukotrienes’ chemical precursors. Polyunsaturated fatty acids can aid in lowering blood triglycerides and cholesterol. They lower the risk factors associated with cardiovascular disease, cancer, and diabetes (type 2). These essential fatty acids are required by the body for brain function and cell growth [40].

### Identification of phospholipids

Twelve phospholipids (Table 2) have been characterized in the methanol extract including mainly, phosphatidic acids (PA), phosphatidylinositols (PI), and phosphatidylglycerols (PG). Structurally, phospholipids are constituted from the esterification of the fatty acid with phosphoglycerol to produce several derivatives. Sometimes, myoinositol, choline, or ethanolamine, may be attached to the phosphate group resulting in a great variation of phospholipids. In the negative-ion mode, the occurrence of carboxylate ions [RCOO]^−^ recognizes the individual fatty acids e.g., *m/z* 277 for octadecatrienoic acid (18:3), 279 for octadecadienoic (18:2), and 255 for hexadecanoic acid (16:1). Moreover, the appearance of some diagnostic ions corresponding to specific functional moieties assists in the characterization of the lipid type, i.e., *m/z* 78 (phosphonate), 152 (phospho-glycerol), and 241 (phospho-myoinositol). For instance, the MS/MS spectrum of PA(18:2/18:2) showed main fragment ions at *m/z* 279 and 152 corresponding to liberation of 18:2 fatty acid and phospho-glycerol units from the parent ion (Table 2). For PI(18:2/16:0) (Fig. S11), it showed a molecular ion at *m/z* 833.5167 with a formula of C_43_H_78_O_13_P^–^, along with the diagnostic ions of phospholipids (171, 152, and 78), other major ions were detected at *m/z* 279 and 255 corresponding to the fatty acid carboxylate anions liberated from *sn1* (less abundant peak) and *sn2* (more abundant peak) positions, respectively [41]. Other characteristic ion was detected at *m/z* 241 corresponding to the inositol-phosphate ion. Following the same fragmentation pattern, the other phospholipids were assigned. Various phospholipids demonstrated antioxidant and anti-inflammatory potentials by suppressing the inflammatory signaling pathways and relieving uncontrolled oxidative stress [42]. To the best of our knowledge, this is the first comprehensive analysis of the metabolites of *T. tipu* flowers to provide chemical-based evidence for their biological potential. Where most of the identified compounds are reputed for their biological effects on human health as antioxidant and anti-inflammatory, that can help to protect body against oxidative stress and inflammatory processes.

**Table 2:** Metabolites identified in the methanolic extract of *T. tipu* flowers using LC–QTOF-MS/MS

ID.	R_t_ (min)	Molecular ion (M-H)^–^	Molecular ion formula	Error ppm	Fragment ions (MS/MS)	Identification
**Sugars and amino acids**						
1	0.92	114.0559	C_5_H_9_NO_2_–	4.87	84, 76, 58	Proline
2	0.96	116.0718	C_5_H_11_NO_2_^–^	-0.31	97, 75, 64, 51	Valine
3	0.97	179.0566	C_6_H_11_O_6_^–^	-3.4	151, 131, 120, 75, 71	Hexose (Glucose)
4	1.08	195.0511	C_6_H_11_O_7_^–^	0.47	129, 87, 75, 56	Gluconic acid
5	1.16	128.0354	C_5_H_6_NO_3_^–^	-0.8	111, 93, 82, 74, 67	Pyroglutamic acid
6	1.18	164.0719	C_9_H_10_NO_2_^–^	-0.97	147, 118, 103, 97, 72	Phenylalanine
7	1.23	130.0872	C_6_H_12_NO_2_^–^	1.81	112, 88, 66	Isoleucine
8	1.45	326.1248	C_15_H_20_NO_7_^–^	-0.86	164, 147	Phenylalanine glucoside
**Organic and phenolic acids**						
9	1.53	115.0034	C_4_H_3_O_4_^–^	0.69	89, 71, 52	Fumaric acid
10	1.79	117.0191	C_4_H_5_O_4_^–^	2.46	99, 73	Succinic acid
11	1.83	133.0143	C_4_H_5_O_5_^–^	-0.66	115, 71	Malic acid
12	1.99	173.0815	C_8_H_13_O_4_^–^	2.59	129, 111, 83, 57	Diethyl succinic acid
13	2.01	191.0197	C_6_H_7_O_7_^–^	0.18	111, 87, 85	Citric acid
14	2.59	191.0568	C_7_H_11_O_6_^–^	2.24	137, 85	Quinic acid
15	2.87	121.0299	C_7_H_5_O_2_^–^	-3.53	121, 92	Benzoic acid
16	3.23	137.0249	C_7_H_5_O_3_^–^	3.38	93, 65	Hydroxy benzoic acid
17	3.36	151.0388	C_8_H_7_O_3_^–^	8.58	139, 137, 129, 119, 109, 107, 102	Methoxy benzoic acid
18	3.89	153.0190	C_7_H_5_O_4_^–^	2.33	109, 108	*Protocatechuic acid*
21	3.96	167.0349	C_8_H_7_O_4_^–^	0.93	152, 108	Vanillic acid
22	4.28	315.0721	C_13_H_15_O_9_^–^	2.19	236, 153, 108	Protocatechuic acid glucoside
19	4.31	329.0876	C_14_H_17_O_9_^–^	2.45	167, 152, 123, 108	Vanillic acid glucoside
20	4.57	341.0879	C_15_H_17_O_9_^–^	2.74	179, 146, 135, 116	Caffeic acid glucoside
23	5.03	295.0456	C_13_H_11_O_8_^–^	1.62	163, 119	Coumaric acid pentoside
24	5.71	325.0914	C_7_H_13_O_7_^–^	-0.22	163, 119	Coumaric acid glucoside
25	6.17	343.1021	C_15_H_19_O_9_^–^	0.59	181, 163, 135, 119	Dihydrocaffeic acid glucoside
26	6.28	147.0655	C_9_H_7_O_2_^–^	0.91	103, 89, 77, 61	Cinnamic acid
27	6.92	193.0494	C_10_H_9_O_4_^–^	-0.84	178, 165, 149, 134, 117	Ferulic acid
28	7.87	353.0873	C_16_H_17_O_9_^–^	1.64	191, 179, 135	Chlorogenic acid
**Coumarins**						
29	6.89	339.0724	C_15_H_15_O_9_^–^	-0.34	177	Esculin
30	9.10	177.0128	C_9_H_5_O_4_^–^	2.84	163, 133, 105, 89, 65	Esculetin
**Flavonoids**						
31	10.02	739.2060	C_40_H_35_O_14_^–^	-2.99	431, 285, 284	Kaempferol 3-*O*-rhamnosyl rutinoside
32	10.05	623.1594	C_28_H_31_O_16_^–^	5.47	315, 300, 271, 243, 151	Isorhamnetin 3-*O*-robinobioside (Narcissin)
33	11.13	609.1435	C_27_H_29_O_16_^–^	3.81	463, 300	Quercetin 3-rutinoside (Rutin)
34	11.14	593.1445	C_28_H_31_O_16_^–^	0.45	431, 285	Kaempferol 3-*O*-rutinoside
35	11.90	577.1569	C_27_H_29_O_14_^–^	-0.47	431, 285, 284, 255	Kaempferol 3,7-di-*O*- rhamnoside (Kaempferitrin)
36	12.37	447.0907	C_21_H_19_O_11_^–^	4.71	357, 339, 327, 297, 285	Luteolin-8-C-glucoside (Orientin)
37	13.42	477.1026	C_22_H_21_O_12_^–^	6.51	315, 314, 300, 151	Isorhamnetin 3-*O*-glucoside
38	13.56	463.0837	C_21_H_19_O_12_^–^	-0.26	300, 271, 255, 179, 151	Quercetin 3-*O*-glucoside (Isoquercetin)
39	13.61	447.0903	C_21_H_19_O_11_^–^	5.97	285, 284, 151	Kaempferol 3-*O*-glucoside (Astragalin)
40	13.79	431.0964	C_21_H_19_O_10_^–^	3.95	341, 311, 283, 269	Apigenin-8-*C*-glucoside (Vitexin)
41	14.95	301.0353	C_15_H_9_O_7_^–^	1.19	179, 151	Quercetin
42	15.08	269.0483	C_15_H_9_O_5_^–^	-8.3	238, 211, 167, 112	Apigenin
**Fatty acids and lipids**						
43	17.82	277.2168	C_18_H_29_O_2_^–^	-0.5	233, 127, 59	Linolenic acid
44	17.99	279.2329	C_18_H_31_O_2_^–^	-0.93	165, 96, 59	Linoleic acid
45	18.08	325.1831	C_18_H_29_O_5_^–^	2.12	237, 121	Trihydroxy octadecatrienoic acid
46	19.22	297.2424	C_18_H_33_O_3_^–^	3.57	279, 261, 239, 183	Hydroxy oleic acid
47	19.31	311.2212	C_18_H_31_O_4_^–^	3.64	223, 183, 87, 57	Octadecendioic acid
48	19.46	327.2909	C_20_H_39_O_3_^–^	-0.70	183	Hydroxy eicosanoic acid
49	19.65	383.3537	C_24_H_47_O_3_^–^	-0.53	337	Hydroxy tetracosanoic acid
50	20.13	593.2729	C_27_H_46_O_12_P^–^	-2.50	277, 241, 152, 78	PI(18:3)
51	20.21	255.2321	C_16_H_31_O_2_^–^	3.51	211, 196	Palmitic acid
52	21.09	507.2719	C_24_H_45_O_9_P	-2.1	279, 227, 152	PG(18:2)
53	21.13	595.2889	C_27_H_48_O_12_P^–^	0.31	279, 241, 152, 78	PI(18:2)
54	21.19	433.2352	C_21_H_38_O_7_P^–^	-1.9	297, 275, 198, 152	PA(18:2)
55	21.28	853.4798	C_45_H_74_O_13_P^–^	-8.7	277, 241, 152	PI(18:3/18:3)
56	21.67	695.4635	C_39_H_68_O_8_P^–^	-2.41	279, 152	PA(18:2/18:2)
57	22.19	825.4540	C_43_H_70_O_13_P^–^	-2.3	277, 259, 249, 241, 152, 78	PI(16:3/18:2)
58	22.40	671.4639	C_37_H_68_O_8_ P^–^	-2.70	279, 255, 152, 78	PA(18:2/16:0)
59	22.79	695.4641	C_39_H_68_O_8_P^–^	-2.30	281, 277, 182, 152	PA(18:3/18:1)
60	23.38	741.4694	C_40_H_70_O_10_P^–^	-3.10	277, 227, 152, 78	PG(16:1/18:3)
61	23.55	833.5167	C_43_H_78_O_13_P^–^	0.52	279, 255, 241, 152, 78	PI(18:2/16:0)
62	24.26	743.4847	C_40_H_72_O_10_P^–^	-3.9	279, 255, 152, 78	PG(18:2/16:1)

*Note* R_t_, retention time; PI, phosphatidyl inositol; PA, phosphatidic acid; PG, phosphatidyl glycerol

#### HPLC-DAD quantification of major identified phenolic compounds in the methanol extract of *T. Tipu* flowers

HPLC-DAD analysis of the methanolic extract of *T. tipu* flowers allowed the identification and quantification of 18 phenolic compounds (phenolic acids, coumarins, and flavonoids). The chromatogram is displayed in Fig. S12. The results are presented in Table 3. The flavonoids: orientin and astragalin illustrated relatively the high abundancies of all identified phenolics corresponding to 2078.88 ± 1.08 and 1898.39 ± 0.81 mg /100 g, respectively. Ferulic acid was the most abundant of all phenolic acids representing 1699.91 ± 1.05 mg /100 g. The coumarin esculetin and its glycoside esculin were quantified representing 77.78 ± 0.55 and 341.80 ± 0.44 mg /100 g, respectively. HPLC-DAD technique allowed further quantification of the identified phenolics, representing their abundances in the methanolic extract. It must be noted that this is the first study quantifying the phenolics of *T. tipu* flowers.

**Table 3:** Quantification of major identified phenolic compounds in the methanol extract of *T. tipu* flowers using HPLC-DAD

Identified compound	Retention time (min)	Conc. (mg /100 g ± SD)
Quinic acid	1.201	259.08 ± 0.90
Benzoic acid	2.311	189.10 ± 0.72
Protocatechuic acid	3.022	179.19 ± 0.62
Vanillic acid	4.802	301.76 ± 0.40
Cinnamic acid	5.675	878.15 ± 0.17
Ferulic acid	6.108	1699.91 ± 1.05
Chlorogenic acid	6.677	92.00 ± 0.71
Esculin	7.656	341.80 ± 0.44
Vitexin	8.019	199.28 ± 0.18
Orientin	8.356	2078.88 ± 1.08
Rutin	9.2	958.92 ± 0.56
Narcissin	9.756	192.14 ± 0.72
Kaempferitrin	10.311	599.95 ± 0.91
Esculetin	11.177	77.78 ± 0.55
Apigenin	12.055	159.23 ± 0.14
Isoquercetin	12.799	462.41 ± 1.73
Astragalin	13.022	1898.39 ± 0.81
Quercetin	14.125	375.66 ± 0.93

SD: standard deviation

### In silico studies

#### Molecular docking

The results of in-vitro biological study revealed that ME induced inhibitory activities against NO, TNF-*α*, IL-*β*, IL-6, NF-KB, and TNF-R2 in LPS-induced RAW264.7 macrophages which was assumed to be associated with its chemical composition. Thus, it is necessary to determine the interactions of the main components of ME of *T. tipu* flowers with Human NOS, COX-2, and 5-LOX active sites *in-silico* using molecular docking and dynamic simulations to ascertain the anti-inflammatory activity and to predict the binding modes and interactions of its major compounds with the active sites of investigated enzymes. Based on the LC-MS profiling, the ME of the flowers was enriched in phenolic compounds, 28 compounds out of the total detected compounds, including 14 phenolic acids, 2 coumarins, and 12 flavonoids. Thus, selected compounds from each phenolic class, including 2 phenolic acids (chlorogenic acid and ferulic acid), 2 coumarins (esculetin and esculin), 4 flavonoids (kaempferol-3-glucoside, narcissin, orientin, and vitexin) were subjected to molecular docking study to investigate their affinity towards the studied enzymes. The results are shown in Table 4. As can be observed, all compounds showed high affinity towards COX-2, with docking scores S ranging from − 8.57 to − 5.05 kcal mol^–1^, 5-LOX (S ranging from − 8.83 to − 5.22 kcal mol^–1^), and Human NOS (S ranging from − 8.23 to − 4.21 kcal mol^–1^). In this context, kaempferol-3-glucoside and orientin exerted the highest docking scores against the targeted enzymes (Table 3). Consequently, they were subjected to further molecular dynamic study.

**Table 4:** Molecular docking data of selected phenolic compounds in the target active sites

Compounds	Hydrogen bonds between atoms of compounds and amino acids of receptor					S- score (binding energy) (kcal/mol)
	Atoms	receptor		Type	Distance (Å)	
		Atoms	Residues			
**Human cyclooxygenase-2**						
Chlorogenic acid	O3	OD1	Asn24	H-donor	3.27	-7.80
	O20	O	Cys21	H-donor	2.82	
Esculetin	O16	OE1	Glu451	H-donor	2.91	-5.05
Esculin	O16	OE1	Glu 451	H-donor	-2.88	-7.37
Ferulic acid	O23	O	Gly30	H-donor	2.94	-5.60
Kaempferol-3-glucoside	O22	N	Cys26	H-acceptor	3.22	-8.57
	O12	O	Gly121	H-donor	2.79	
	O24	OD2	Asp111	H-donor	2.86	
	O39	OE1	Glu451	H-donor	-2.6	
	O43	O	Glu451	H-donor	-1.8	
	6-ring	CG	Pro139	Pi-H	4.15	
Narcissin	O47	SG	Cys26	H-donor	4.01	-5.13
	O47	O	Cys26	H-donor	2.64	
Orientin	O40	O	Pro140	H-donor	2.80	-8.11
	O52	SG	Cys145	H-donor	3.80	
Vitexin	O5	ND2	Asn19	H-acceptor	3.26	-7.05
	O34	N	Cys32	H-acceptor	2.83	
**Human 5-lipoxygenase**						
Chlorogenic acid	O20	OE1	Gln 141	H-donor	2.79	-7.55
	O23	O	Val110	H-donor	3.07	
Esculetin	O18	O	Val 110	H-donor	2.88	-5.22
	O3	NZ	Lys394	H-acceptor	2.91	
Esculin	O39	O	Val 110	H-donor	2.71	-6.81
	O3	N	Asp166	H-acceptor	3.30	
Ferulic acid	O23	O	Pro 98	H-donor	3.01	-5.29
Kaempferol-3-glucoside	O35	OE2	Glu134	H-donor	2.66	-8.83
	O39	NH1	Arg101	H-acceptor	3.40	
	O39	N	Val 110	H-acceptor	3.23	
Narcissin	O43	OE1	Gln141	H-donor	2.74	-5.74
	O47	OD2	Asp166	H-donor	2.88	
Orientin	O22	O	Val107	H-donor	2.70	-8.58
	O36	N	Val110	H-acceptor	2.97	
Vitexin	O25	O	Trp102	H-donor	3.14	-6.13
	O78	CD	Arg138	H-acceptor	3.44	
**Human Nitric oxide synthase (NOS) enzymes synthesize**						
Chlorogenic acid	O26	SG	Cys200	H-donor	3.43	-5.76
Esculetin	O16	O	Asn370	H-donor	2.84	-4.21
Esculin	O16	OG	Ser242	H-donor	3.16	-5.80
	O27	OE2	Glu377	H-donor	2.83	
Ferulic acid	6-ring	CB	Arg199	Pi-H	4.48	-5.10
Kaempferol-3-glucoside	O39	O	Trp372	H-donor	2.95	-8.23
	O43	OE2	Glu377	H-donor	2.95	
	O51	N	Ile201	H-acceptor	3.22	
	6-ring	CB	CYS200	pi-H	4.03	
	6-ring	6-ring	PHE369	pi-H	3.86	
	6-ring	6-ring	TRP194	pi-H	3.69	
Narcissin	O75	OH	Tyr347	H-acceptor	3.07	-7.54
Orientin	O40	OE2	Glu377	H-donor	2.95	-7.92
	O44	OE2	Glu377	H-donor	2.95	
	C2	6-ring	Tyr489	H-Pi	4.71	
	6-ring	5-ring	Trp194	Pi-pi	3.91	
	6-ring	5-ring	Trp194	Pi-pi	3.82	
Vitexin	O24	OG	Ser242	H-donor	2.95	-6.23
	O42	OE2	Glu377	H-donor	3.00	

### Molecular dynamic (MD) simulations

**Fig. 3:**
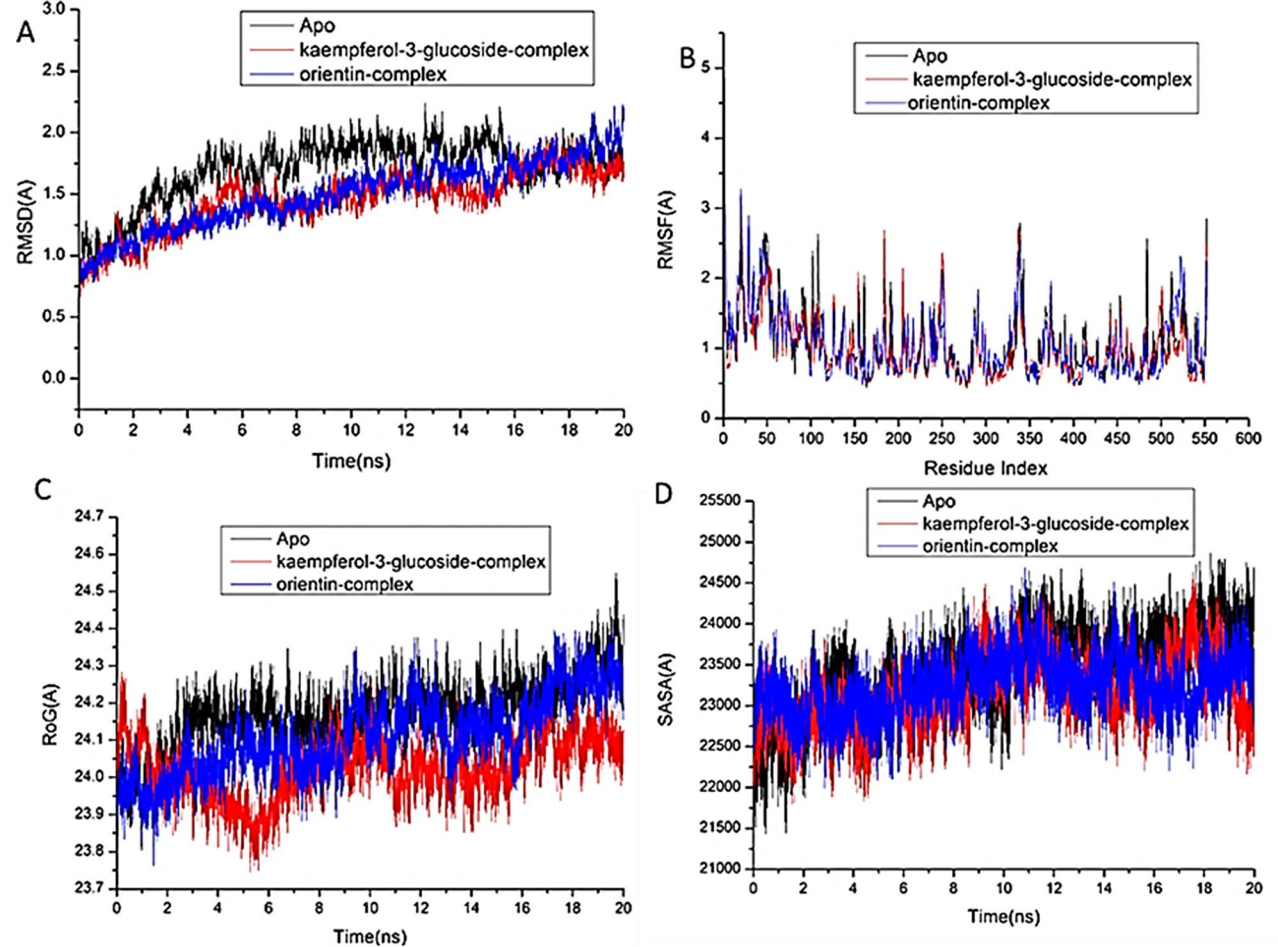
[**A**] RMSD of the protein backbone’s Cα atoms. [**B**] RMSF of each residue of the protein backbone Cα atoms of protein residues (**C**) ROG of Cα atoms of protein residues; (**D**) solvent accessible surface area (SASA) of the Cα of the backbone atoms relative (black) to the starting minimized over 20 ns for the catalytic domain binding site of cyclooxygenase-2 enzyme with kaempferol-3-glucoside complex system (red), and orientin complex system (blue)

**Fig. 4:**
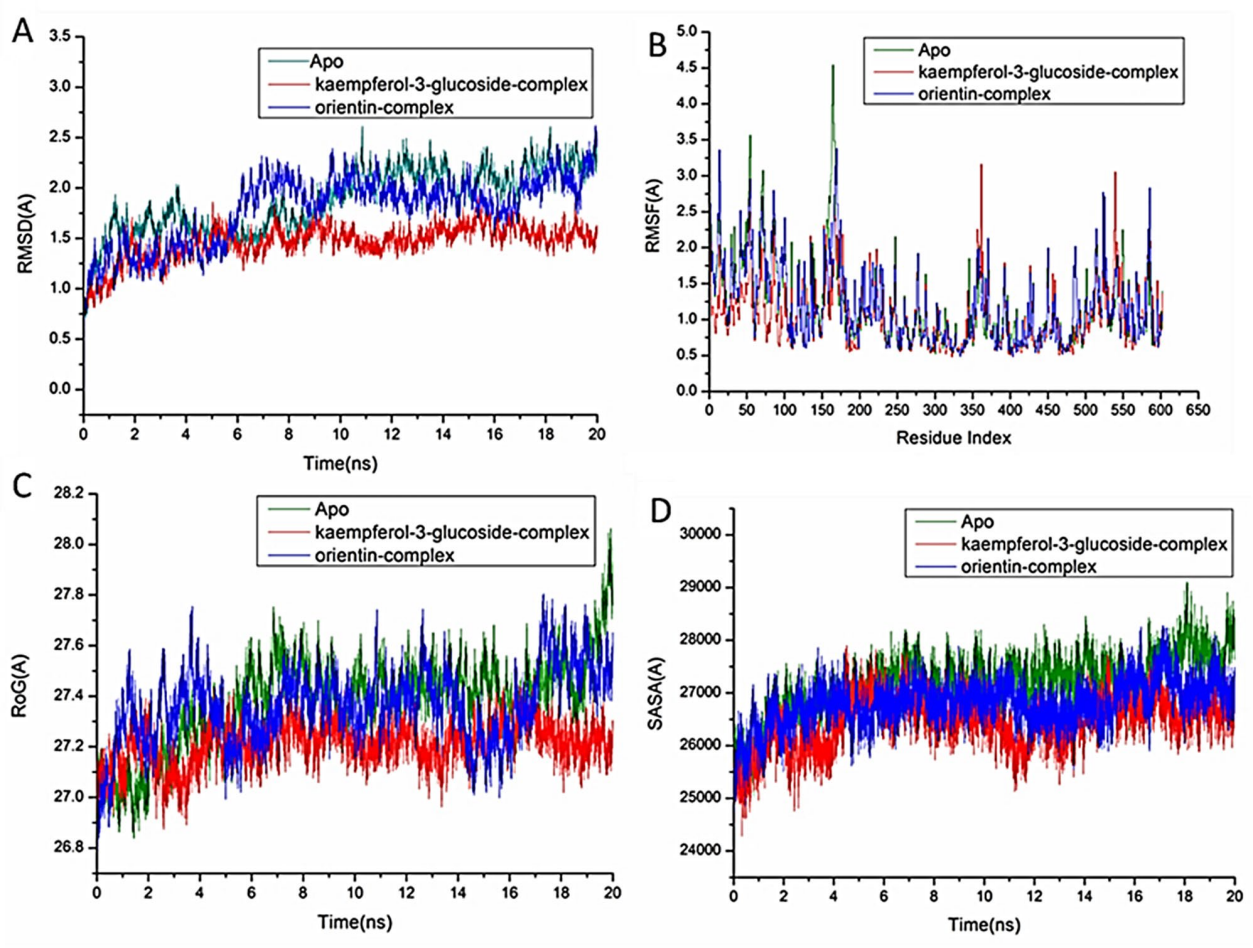
[**A**] RMSD of the protein backbone’s Cα atoms. [**B**] RMSF of each protein residue’s Cα atom; (**c**) ROG of each residue’s Cα atom; (**d**) solvent accessible surface area (SASA) of the backbone atoms relative to the starting minimized over 20 ns for the catalytic domain binding site of 5-lipoxygenase with kaempferol-3-glucoside complex system (red), and orientin complex system (blue)

**Fig. 5:**
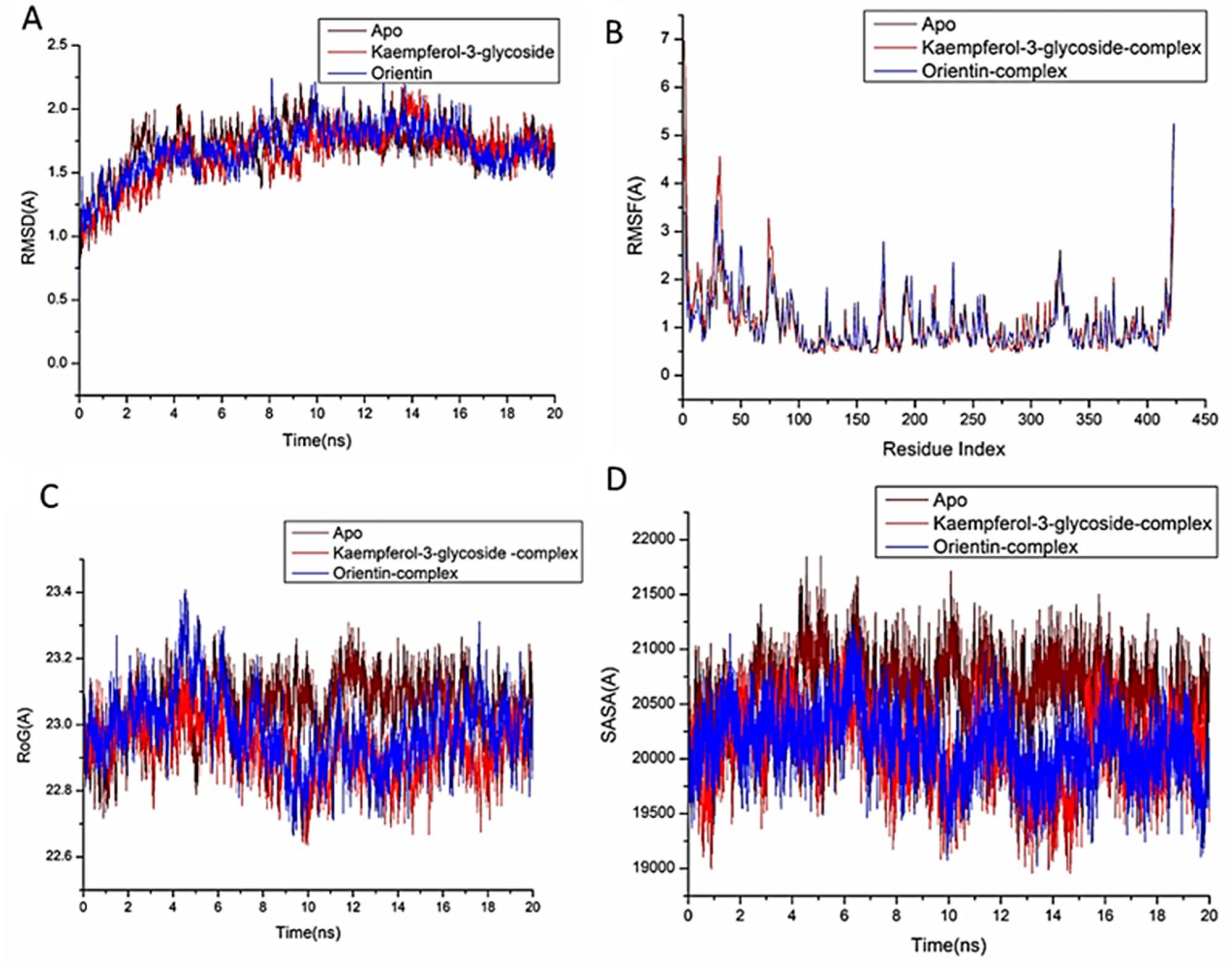
[**A**] RMSD of the protein backbone’s Cα atoms. [**B**] RMSF of each residue of the protein backbone Cα atoms of protein residues (**c**) ROG of Cα atoms of protein residues; (**d**) solvent accessible surface area (SASA) of the Cα of the backbone atoms relative (black) to the starting minimized over 20 ns for the catalytic domain binding site of Human Nitric oxide synthase with kaempferol-3-glucoside complex system (red), and orientin complex system (blue)

In the study of biological systems, Molecular Dynamic (MD) integration simulations enable investigation of the physical motion of atoms and molecules that cannot be easily accessed by any other means. The insight obtained from performing this simulation offers an extensive viewpoint on the dynamical evolution of biological systems, including changes in conformation and molecular interaction [43]. Therefore, this simulation was run to predict how the selected compounds would behave once they bound to the protein active site, as well as their interaction and stability [44, 45]. For tracing erratic motions and eliminating artefacts that might appear during the simulation, system stability must be validated. This study used Root-Mean-Square Deviation (RMSD) to analyze system stability during the 20 ns simulations. The recorded the average RMSD values for the entire frames of the systems were 1.69 ± 0.25Å, 1.45 ± 0.24Å, and 1.49 ± 0.27Å, for Apo-COX protein, kaempferol-3-glucoside-COX, and orientin–COX complex systems (Fig. 3A), 1.87 ± 0.32Å, 1.46 ± 0.17Å, and 1.79 ± 0.33Å for Apo-LOX protein, kaempferol-3-glucoside-LOX, and orientin–LOX complex systems, respectively (Fig. 4A), and 1.69 ± 0.18Å, 1.65 ± 0.21Å, and 1.68 ± 0.19 Å for Apo-NOS protein, kaempferol-3-glucoside- NOS, and orientin–NOS complex systems, respectively (Fig. 5A).

For the purpose of analyzing residue behavior and its relationship to the ligand during MD simulation, it is essential to evaluate protein structural flexibility upon ligand binding [46]. Using the Root-Mean-Square Fluctuation (RMSF) technique, protein residue variations were assessed to determine the impact of inhibitor binding to the relevant targets across 20 ns of simulations. The computed average RMSF values for the entire frames of the systems were 1.05 ± 0.44Å, 1.01 ± 0.44Å, and 1.05 ± 0.49 Å, for Apo-COX protein, kaempferol-3-glucoside-COX, orientin–COX complex systems (Fig. 3B), 1.18 ± 0.57Å, 1.05 ± 0.44Å, and 1.18 ± 0.54Å for Apo-LOX protein, kaempferol-3-glucoside-LOX, orientin–LOX complex systems, respectively (Fig. 4B), and 1.11 ± 0.66Å,1.05 ± 0.64Å, and 1.07 ± 0.60 Å for Apo-NOS protein, kaempferol-3-glucoside-NOS, and orientin–NOS complex systems, respectively (Fig. 5B). These values revealed that the ligand bound to protein kaempferol-3-glucoside-complex system has a lower residue fluctuation than the other systems.

During an MD simulation, ROG was chosen to assess the stability and overall compactness of the system [47, 48].The average Rg values for the entire frames of the systems were 24.17 ± 0.09Å, 24.02 ± 0.08 Å, and 24.10 ± 0.10 Å, for Apo-COX protein, kaempferol-3-glucoside-COX, and orientin–cox complex systems (Fig. 3C), 27.37 ± 0.17Å, 27.21 ± 0.08Å, and 27.36 ± 0.14Å for Apo-LOX protein, kaempferol-3-glucoside-LOX, and orientin–LOX complex systems, respectively (Fig. 4C). 23.05 ± 0.08Å, 22.92 ± 0.08Å, and 22.98 ± 0.18 Å for Apo-NOS protein, kaempferol-3-glucoside-NOS, orientin–NOS complex systems, respectively (Fig. 5C). According to the observed behavior, kaempferol-3-glucoside- bound complex has a highly stiff structure against three enzymes.

By calculating the protein’s solvent accessible surface area (SASA), the hydrophobic core compactness of the protein was assessed. This was done by measuring the protein’s solvent-visible surface area, which is crucial for biomolecule stability [49]. The average SASA values for the complete frames of the Apo-COX protein, kaempferol-3-glucoside-COX, and orientin-COX complex systems were 23,437, 23,156, and 23243.86, respectively (Fig. 3D). 27152Å, 26451Å, and 26796.51Å, for Apo-LOX protein, kaempferol-3-glucoside-LOX, orientin–LOX complex systems, respectively (Fig. 4D). 20645Å, 20053.87Å, and 20125.54Å for Apo-NOS protein, kaempferol-3-glucoside-NOS, and orientin–NOS complex systems, respectively (Fig. 5D).

When paired with the data from the RMSD, RMSF, and ROG computations, the SASA finding revealed that the kaempferol-3-glucoside and orientin complex systems remain intact inside the catalytic binding site for the three enzymes.

### Binding interaction mechanism based on binding free energy calculation

A well-known method for determining the free binding energies of small molecules to biological macromolecules is the molecular mechanics energy methodology (MM/GBSA), which combines the generalized Born and surface area continuum solvation [50]. The MM-GBSA program in AMBER18 was used to determine the binding free energies using snapshots taken from the systems’ trajectories. Except for Gsolv, all of the estimated energy components (Table 5) showed large negative values indicating favorable interactions.

**Table 5:** The calculated energy binding for the selected compounds

Energy Components (kcal/mol)					
**Human cyclooxygenase-2**					
Complex	**ΔE**_**vdW**_	**ΔE**_**elec**_	**ΔG**_**gas**_	**ΔG**_**solv**_	**ΔG**_**bind**_
Kaempferol-3-glucoside	-57.06 ± 0.18	-33.40 ± 0.63	-90.50 ± 0.61	40.69 ± 0.37	-49.81 ± 0.31
Orientin	-57.98 ± 0.16	-26.44 ± 0.41	-64.43 ± 0.58	33.84 ± 0.25	-37.29 ± 0.24
**Human 5-lipoxygenase**					
Complex	**ΔE**_**vdW**_	**ΔE**_**elec**_	**ΔG**_**gas**_	**ΔG**_**solv**_	**ΔG**_**bind**_
Kaempferol-3-glucoside	-44.39 ± 0.25	-67.54 ± 0.36	-111.93 ± 0.38	55.62 ± 0.25	-56.31 ± 0.24
Orientin	-53.08 ± 0.23	-25.47 ± 0.46	-78.56 ± 0.39	35.33 ± 0.29	-43.22 ± 0.19
**Human Nitric oxide synthase (NOS)**					
Complex	**ΔE**_**vdW**_	**ΔE**_**elec**_	**ΔG**_**gas**_	**ΔG**_**solv**_	**ΔG**_**bind**_
Kaempferol-3-glucoside	-44.62 ± 0.58	-70.47 ± 0.33	-115.10 ± 0.15	67.53 ± 0.93	-47.56 ± 0.61
Orientin	-35.37 ± 0.63	-60.79 ± 0.73	-96.17 ± 0.71	65.02 ± 0.25	-31.14 ± 0.74

∆EvdW = van der Waals energy; ∆Eele = electrostatic energy; ∆Gsolv = solvation free energy; ∆Gbind = calculated total binding free energy

### Identification of the critical residues responsible for ligands binding

**Fig. 6:**
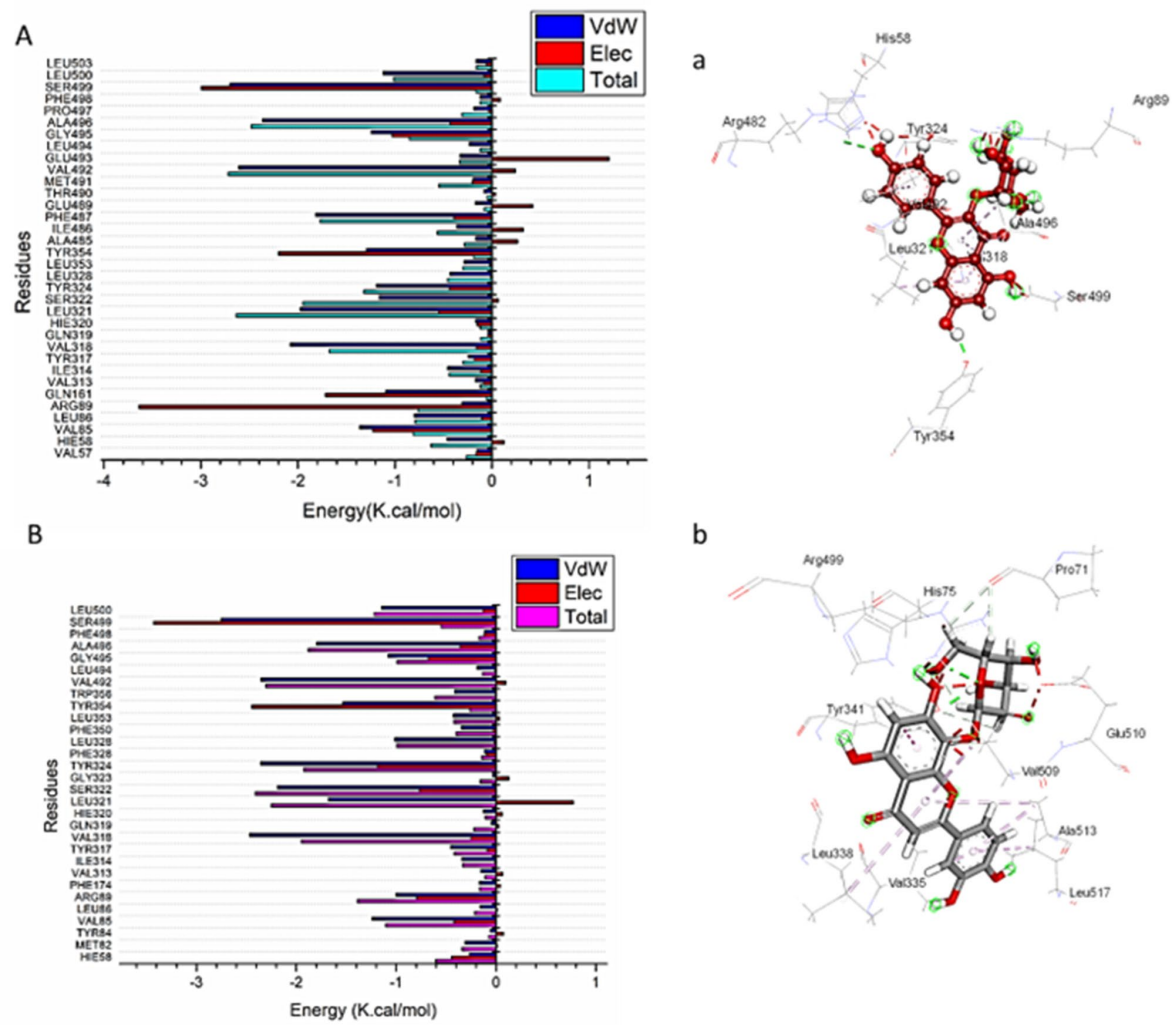
Per-residue decomposition plots showing the energy contributions to the binding and stabilization of kaempferol-3-glucoside and orientin into the catalytic binding site of Human cyclooxygenase-2 [**A**], [**B**] Corresponding inter-molecular interactions are shown [**a**], [**b**]

To understand more about key residues involved in the inhibition of the catalytic binding site of COX-2 receptor, the total energy involved when substance contacts these enzymes was further broken down into the participation of specific site residues. From Fig. 6A, the major favorable contribution of kaempferol-3-glucoside to the COX-2 binding site receptor is mainly observed from residues Val57 (-0.154 kcal/mol), Hie58 (-0.463 kcal/mol), Val85 (-1.362 kcal/mol), Leu86 (-0.799 kcal/mol), Arg89 (-0.308 kcal/mol), Gln161 (-1.095 kcal/mol), Ile314 (-0.456 kcal/mol), Tyr317 (-0.24 kcal/mol), Val318 (-2.078 kcal/mol), Leu321 (-1.975 kcal/mol), Ser322 (-1.165 kcal/mol), Tyr 324 (-1.19 kcal/mol), Leu328 (-0.432 kcal/mol), Leu353 (-0.288 kcal/mol), Tyr354 (-1.295 kcal/mol), Ile486 (-0.356 kcal/mol), Phe 487 (-1.81 kcal/mol), Val492 (-2.61 kcal/mol), Gly495 (-1.245 kcal/mol), and Ala496 (-2.36 kcal/mol).

Regarding orientin, the major favorable contribution to the COX-2 binding site receptor is principally observed from residues Hie58 (-0.267 kcal/mol), Met82 (-0.311 kcal/mol), Val 85 (-1.239 kcal/mol), Ile314 (-0.341 kcal/mol), Tyr317 (-0.455 kcal/mol), Val318 (-2.468 kcal/mol), Leu321 (-1.68 kcal/mol), Ser 322 (-2.189 kcal/mol), Tyr324 (-2.357 kcal/mol), Leu328 (-1.015 kcal/mol), Phe350 (-0.348 kcal/mol), Leu353 (-0.427 kcal/mol), Tyr354 (-1.535 kcal/mol), Trp356 (-0.418 kcal/mol), Val492 (-2.354 kcal/mol), Gly495 (-1.08 kcal/mol), Ala496 (-1.794 kcal/mol), and Ser499 (-2.752 kcal/mol) (Fig. 6B).

**Fig. 7:**
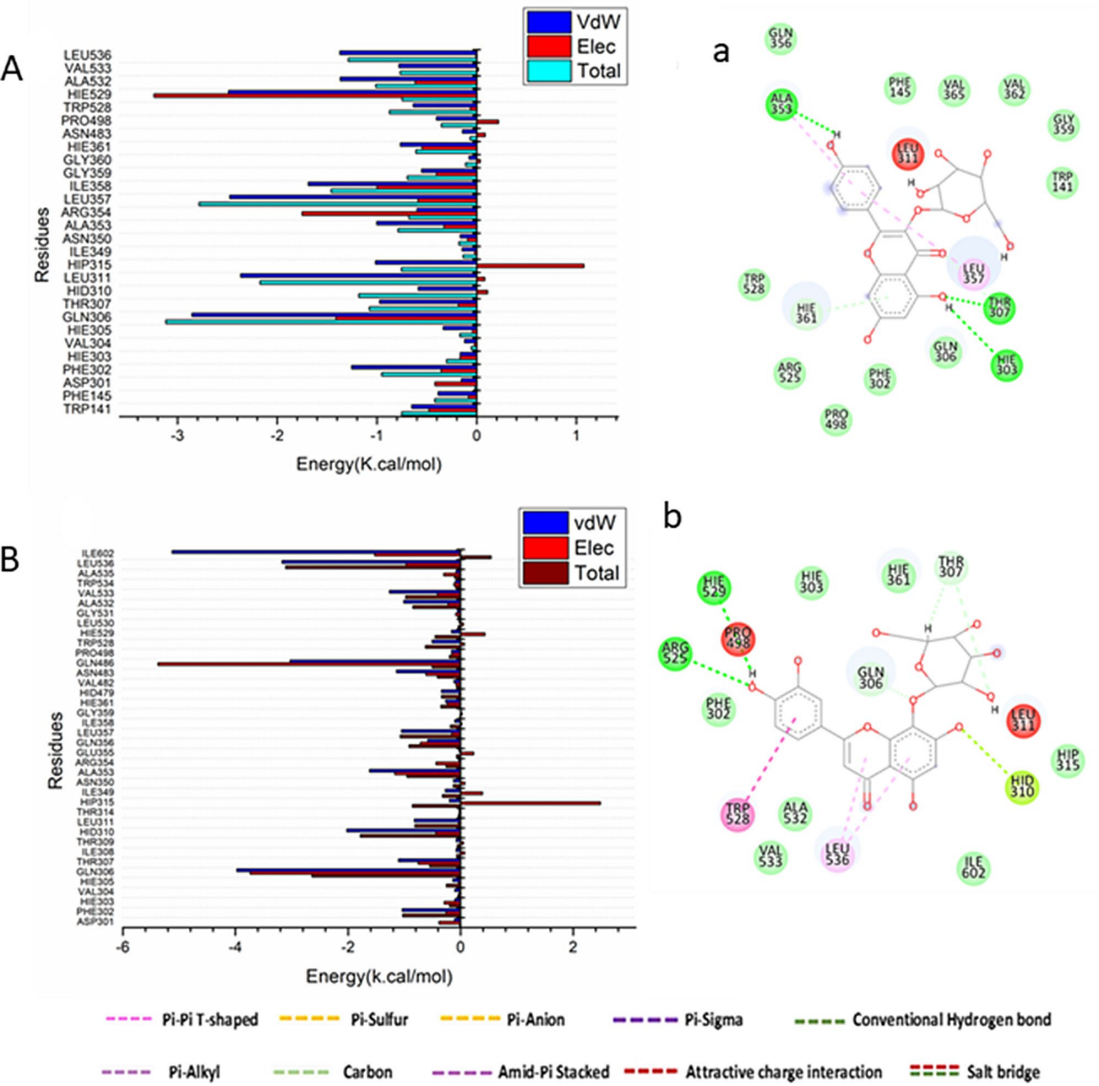
Per-residue decomposition plots showing the energy contributions to the binding and stabilization of kaempferol-3-glucoside and orientin into the catalytic binding site of Human-5-lipoxygenase [**A**], [**B**]. Corresponding inter-molecular interactions are shown [**a**], [**b**]

From Fig. 7A, the major favorable contribution of kaempferol-3-glucoside to the 5-lipoxygenase binding site receptor is predominantly observed from residues Trp141 (-0.652 kcal/mol), Phe145 (-0.387 kcal/mol), Phe 302 (-1.255 kcal/mol), Gln306 ( -2.856 kcal/mol), Thr307 (-0.975 kcal/mol), Hid310 (-0.588 kcal/mol), Leu311 (-2.367 kcal/mol), Hip315 (-1.015 kcal/mol), Ala353 (-1.003 kcal/mol), Arg354 (-0.6 kcal/mol), Leu357 (-2.475 kcal/mol), Ile358 (-1.68 kcal/mol), Gly359 (-0.552 kcal/mol), Hie361 (-0.766 kcal/mol), Pro 498 (-0.405 kcal/mol), Trp528 (-0.637 kcal/mol), Hie529 (-2.488 kcal/mol), Ala532 (-1.369 kcal/mol), Val533 (-0.78 kcal/mol), and Leu356 (-1.372 kcal/mol). Alternatively, the major favorable contribution of orientin to the 5-lipoxygenase binding site receptor is mostly detected from residues Phe302 (-1.036 kcal/mol), Gln306 (-3.975 kcal/mol), Thr307 (-1.105 kcal/mol), Hid310 (-2.021 kcal/mol), Leu311 (-0.822 kcal/mol), Ala353 (-1.617 kcal/mol), Gln356 (-0.585 kcal/mol), Hid479 (-0.338 kcal/mol), Asn483 (-1.139 kcal/mol), Gln486 (-3.03 kcal/mol), Trp528 (-0.507 kcal/mol), Ala532 (-1.011 kcal/mol), Val533 (-1.265 kcal/mol), Leu536 (-3.171 kcal/mol), and Ile602 (-5.123 kcal/mol) (Fig. 7B).

**Fig. 8:**
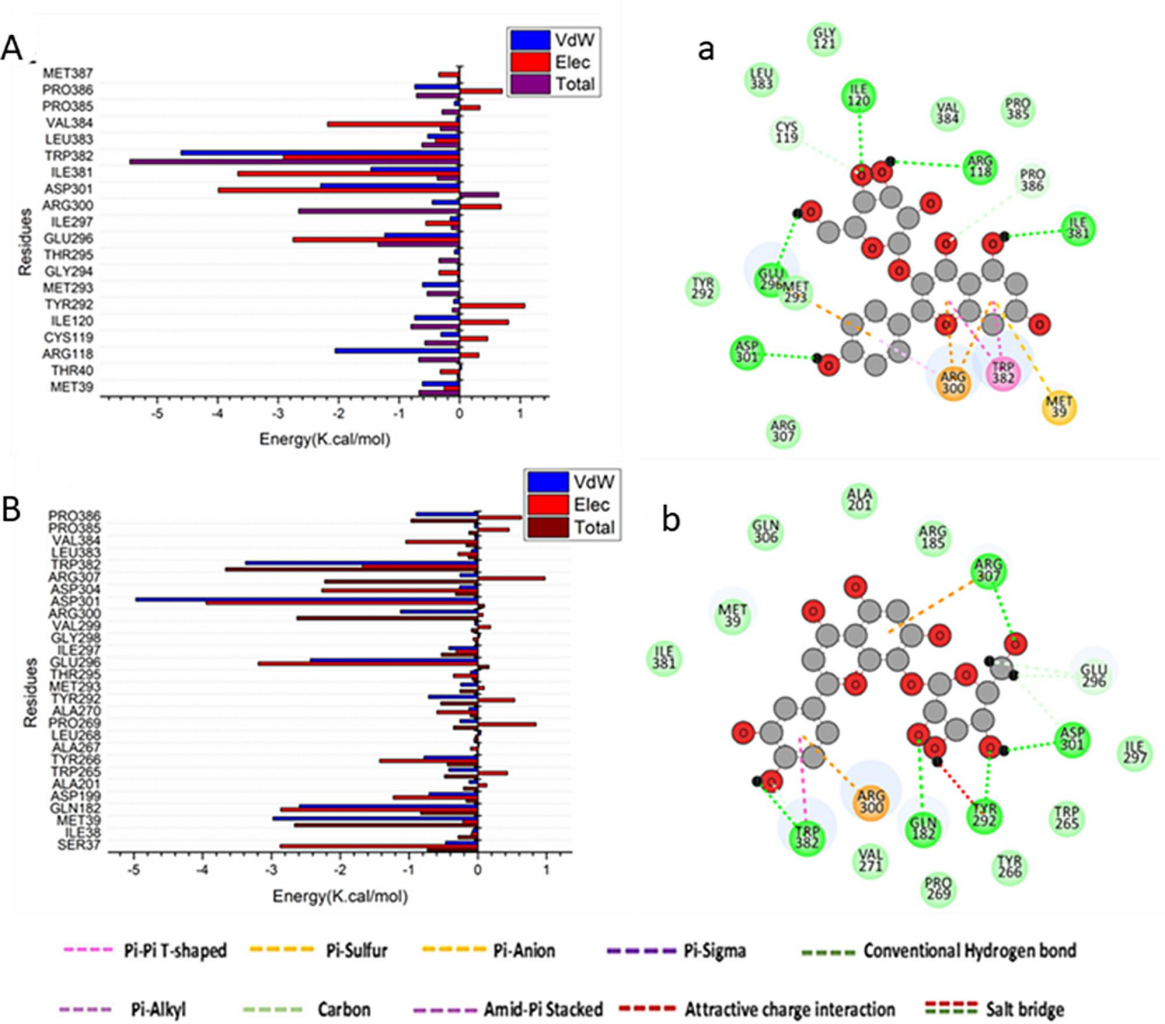
Per-residue decomposition plots showing the energy contributions to the binding and stabilization of kaempferol-3-glucoside and orientin into the catalytic binding site of Human Nitric oxide synthase (NOS) protein [**A**], [**B**]. Corresponding inter-molecular interactions are shown [**a**], [**b**]

Finally, from Fig. 8A, the major favorable contribution of kaempferol-3-glucoside to the Human Nitric oxide synthase (NOS) synthesize enzymes protein binding site receptor is predominantly observed from residues Met39 (-0.616 kcal/mol), Arg118 (-2.059 kcal/mol), Cys119 (-0.312 kcal/mol), Ile120 (-0.745 kcal/mol), Met293 (-0.613 kcal/mol), Glu296 (-1.238 kcal/mol), Arg300 (-0.455 kcal/mol), Asp301 (-2.298 kcal/mol), Ile381 (-1.47 kcal/mol), Trp382 (-4.6 kcal/mol), Leu383 (-0.527 kcal/mol), and Pro386 (-0.741 kcal/mol). On the other, the major favorable contribution of orientin to the Human Nitric oxide synthase (NOS) protein binding site receptor is predominantly observed from residues Ser37 (-0.468 kcal/mol), Met39 (-2.975 kcal/mol), Gln182 (-2.593 kcal/mol), Asp199 (-0.709 kcal/mol), Trp265 (-0.422 kcal/mol), Tyr266 (-0.782 kcal/mol), Pro269 (-0.259 kcal/mol), Tyr292 (-0.716 kcal/mol), Met293 (-0.249 kcal/mol), Glu296 (-2.435 kcal/mol), Ile297 (-0.42 kcal/mol), Arg300 (-1.125 kcal/mol), Asp301 (-4.969 kcal/mol), Asp304 (-0.265 kcal/mol), Arg307 (-0.26 kcal/mol), Trp382 (-3.375 kcal/mol), and Pro386 (-0.896 kcal/mol) (Fig. 8B). Interestingly, the docking results come in agreement with the previous reports demonstrated the antioxidant and anti-inflammatory activities of orientin and kaempferol-3-glucoside [51, 52].

## Conclusion

This investigation revealed the promising efficiency of *T. tipu* flowers as antioxidant and anti-inflammatory drug, which is obviously related to its chemical profile and as indicated by the computational analysis. The results suggested the useful incorporation of the flowers’ extract in pharmaceutical and cosmetic industries owing to its potential to suppress the inflammatory key enzymes (iNOS, COX-2, and 5-LOX), as well as scavenge free radicals through its high antioxidant properties. However, it should be noted that these findings are preliminary, and further research with the methanol extract or after purification of specific compounds, followed by more detailed and conclusive in vivo and clinical studies, is strongly advised to fully exploit *T. tipu* flowers’ potential in the pharmaceutical industry.

### Electronic supplementary material

Below is the link to the electronic supplementary material.

Supplementary Material 1

## Data Availability

All data generated or analyzed during this study are included in this published article and its supplementary information file.

## Abbreviations

5-LOX: 5-lipoxygenase
ABTS, 2,2': azino-di(3-ethylbenzthiazoline-6-sulfonic acid
COX-2: cyclooxygenase 2
DPPH, 2,2: diphenyl-1-picrylhydrazyl
FRAP: ferric reducing antioxidant power
IC_50_: half maximal inhibitory concentration
IL: interleukin; iNOS: inducible nitric oxide synthase
LC-QTOF-MS/MS: liquid chromatography-quadrupole-time-of-flight-mass spectrometry
LPS: lipopolysaccharide
MD: molecular dynamic
ME: methanolic extract
MTT: (3-[4,5-dimethylthiazol-2-yl]-2,5 diphenyl tetrazolium bromide)
NF-KB: Nuclear factor kappa B
NO: nitric oxide
TE: Trolox equivalent
TNF-α: tumor necrosis factor alpha
TNF-R2: tumor necrosis factor receptor 2
