# Supplementary material for: LC-MS/MS profiling of Tipuana tipu flower, HPLC-DAD quantification of its bioactive components, and interrelationships with antioxidant, and anti-inflammatory activity: in vitro and in silico approaches
Source: BMC Complement Med Ther. 2024 Apr 26;24:176. doi: 10.1186/s12906-024-04467-5 (PMC11055345; doi:10.1186/s12906-024-04467-5)
Supplement: Supplementary file 1 — Supplementary Material 1 [file 12906_2024_4467_MOESM1_ESM.docx]

**Supplementary Material**

**LC-MS/MS profiling of *Tipuana tipu* flower****, HPLC-DAD quantification of its bioactive components, and interrelationships with antioxidant, and anti-inflammatory activity: *in vitro* and *in silico* approaches**

*Rana M. Ibrahim^1,^†, Passent M. Abdel-Baki^1,^†^,*^, Ahmed A. El-Rashedy^2^, Nariman E. Mahdy^1^*

***^1^****Pharmacognosy Department, Faculty of Pharmacy, Cairo University, Kasr-El-Ainy Street, 11562, Cairo, Egypt*

***^2^****Natural and Microbial Products Department, National Research Center (NRC), Dokki 12622, Giza, Egypt.*

********Corresponding author: Passent M. Abdel-Baki,* [*passent.mohamed@pharma.cu.edu.eg*](mailto:passent.mohamed@pharma.cu.edu.eg)

*† These authors contributed equally to this work.*

**Materials and Methods**

**Chemicals and reagents**

Lipopolysaccharide (LPS) and 3-(4,5-dimethylthiazol-2-yl)-2,5-diphenyl-2H-tetrazolium bromide (MTT) were obtained from Sigma-Aldrich Chemicals, Germany. IL-*β* (ab46052), IL-6 (ab178013), and TNF-*α* (ab181421) kits were obtained from (abcam®, Human ELISA kit; China). The absorbances were determined using a Tecan microplate reader (Infinite F50, Switzerland). The standards used in HPLC and the reagents used for biological investigations were supplied by Sigma Chemical Company (CA, USA). **Antioxidant assays**

The extracts were prepared in dimethyl sulfoxide (DMSO) at a concentration of 1mg/mL. Each assay was done in triplicate. Serial dilutions of Trolox (6-hydroxy-2,5,7, 8-tetramethylchroman-2-carboxylic acid) were prepared (50–800 μM) to establish dose-response curves for each assay. Ascorbic acid was served as an antioxidant standard, as previously described [1]. The absorbances were measured using Tecan, microplate reader, (Infinite F50, Switzerland). The results were expressed as micromolar Trolox equivalent per gram of dry extract (μM TE/g) and recorded as mean ± SD.

**DPPH assay**

The DPPH (2, 2-diphenyl-1-picrylhydrazyl) assay was conducted according to previously published procedure [2]. Thirty µL of the tested sample was added to 270 µL of DPPH methanolic solution (6x10^-5^ mol/L). A blank was formed of 300 µL of DPPH methanolic solution.

**ABTS assay**

The ABTS (2,2`-Azino-bis(3-ethylbenzothiazoline-6-sulfonic acid)) assay was done in accordance to previously published method [3].

ABTS radical cation (ABTS•+) was generated by the reaction of ABTS aqueous solution (5 mL, 7 mM) and potassium persulfate (88 µL, 2.45 mM). The generated ABTS•+ solution was diluted with methanol to an absorbance of 0.700 at 750 nm, and a 1 mL aliquot was combined with 10 μL of each tested sample solution. The decrease of the formed ABTS color intensity was determined spectrophotometrically at 750 nm. The blank was made up of 200 μL of methanol.

**FRAP assay**

The FRAP assay was designed according to the previous procedures [4]. A blue-colored ferrous-probe complex from a colorless ferric-probe complex was formed due to the reduction of Fe^3^+ to Fe^2^+, which is chelated by 2,4,6-tris(2- pyridyl)-s-triazine (TPTZ) forming Fe2+-TPTZ complex. The FRAP reagent was prepared by mixing 10 volumes of 300 mM sodium acetate buffer (pH 3.6) with 1 volume of 10 mM 2,4,6-tri(2-pyridyl)-s-triazine (TPTZ) in 40 mM hydrochloric acid and 1 volume of 20 mM ferric chloride. The tested samples (100 μL) and deionized water (300 μL) were incubated with 3 mL of freshly prepared FRAP reagent at 37 °C for 6 min. The resulting blue colour was determined at 593 nm at the end of the incubation time.

**Anti-inflammatory activity**

***In vitro* cyclooxygenase (COX-2) inhibitory activity**

Stock solutions of the tested extracts (1 mg/mL) were prepared in DMSO. Subsequent serial dilutions of the extracts (125-0.98 µg/mL), as well as the standard (250-0.98 µM), were done in a 96-well plate. The inhibition of human recombinant COX-2 enzyme (Cayman Chemicals, USA) was assessed colorimetrically [5]. Blank was done by the reaction mixture containing assay buffer and heme. The 100% initial activity (IA) was established by a mixture of COX-2 assay buffer and heme. Celecoxib served as a positive control. The absorbances of oxidized TMPD (blue color) were determined in ELISA plate reader at 590 nm. The % inhibition was determined according to the following formula:

COX % Inhibition= [(100%IA−Inhibitor)/ 100% IA x 100]. Where IA: 100% initial activity.

The concentration at which the tested sample produces 50% inhibition of COX-2 (IC_50_) was determined.

***In vitro* lipoxygenase (LOX) inhibitory activity**

The LOX inhibitory activity was assessed colorimetrically according to a published method [6] depending on enzymatic oxidation of linoleic acid to the corresponding hydroperoxide. A serial dilution of each tested extract (125- 0.98 µg/mL) was prepared in DMSO. The reaction mixture was formed of substrate (50 μM linoleic acid in 0.2 M borate buffer pH 9), 0.9 nM 5-LOX (MyBioSource, USA) and different concentrations of the tested samples. To obtain the 100% initial activity of LOX, DMSO was used as a solvent control instead of the tested sample. A reference drug was used (Zileuton). The absorbances were recorded at 234nm. The % inhibition was calculated using the following formula:

LOX % Inhibition= [(100%IA−Inhibitor)/ 100% IA x 100]. Where IA: 100% initial activity.

The IC_50_ of each extract was determined.

**Cell culture of RAW264.7 macrophages**

The RAW264.7 macrophage cells (Shanghai BOGO Industrial Co., Ltd., Shanghai, China) were cultured on Dulbecco’s modified Eagle’s medium (DMEM), supplemented with 10% fetal bovine serum (Hyclone, General Electric Healthcare Life Sciences, Mississauga, Canada) and 1% penicillin streptomycin (Solarbio Life Sciences, Beijing, P.R. China) at 37 °C and 5% CO_2_. The cells were utilized in the assays at 60% confluency after 24 hours.

**Cell viability assay**

The cell viability assay was assessed using MTT reduction assay [7]. The RAW264.7 macrophages (1 × 10^16^ cells per well) were incubated for 24 hours. Two hundred µL of the methanolic extract serial dilution (0-1000 μg/mL) replaced the culture medium, followed by incubation for 24 h, then replaced by 90 µL of fresh culture medium. In each well, 5 mg/mL of 3-(4,5-dimethylthiazol-2-yl)-2,5-diphenyl-2H-tetrazolium bromide (MTT; Sigma-Aldrich Chemicals, Germany) in phosphate buffer (PBS, pH 7.4) was added and incubated for 5 h at 37 °C (5% CO2). The unreacted dye was eluted, and 200 µL/well DMSO was added to dissolve the formazan crystals. The absorbances were recorded at 570 nm.

**Extracts treatment**

A stock solution of the methanolic extract (1 mg/mL) was made by dissolving it in 0.1% DMSO in PBS (pH 7.2). Then it was further diluted with Dulbecco's Modified Eagle Medium (DMEM) to obtain the desired concentration. The RAW264.7 macrophages were seeded (4 × 10^3^ cells per well) overnight, washed with DMEM, and then pre-treated with different concentrations of the methanolic extract (125- 0.98 µg/mL), and the standard drug (125 - 0.06 µM) for 30 min. The macrophages were further incubated with 1 μg/mL lipopolysaccharide (LPS; Sigma-Aldrich Chemicals, Germany) for 24 h [8]. For negative control LPS-, cells were treated with DMSO (0.1%). The RAW 26.7 cell monolayers were washed with ice-cold PBS (pH 7.2), trypsinized, then the lysis buffer (1% Triton X-100, 50 mMTris–HCl, pH 7.4, 0.2% sodium dodecyl sulfate, 0.2% sodium deoxycholate, 1 mM phenylmethylsulfonyl fluoride, 1 mM sodium ethylenediaminetetraacetate, 5 μg/mL of leupeptin, 5 μg/mL of aprotinin) was added. The supernatant was collected by centrifugation (5000 rpm for 10 min). For anti-inflammatory assays, the cell lysates were kept at − 80 °C.

**Inhibitory activity of the methanolic extract (ME) on inducible nitric oxide synthase (iNOS) activity in LPS stimulated RAW 264.7 macrophages**

In each well, equal volumes (100 µL) of each cell lysate and iNOS assay buffer (1×) were mixed. Then the mixtures were incubated for 2 hours at 37◦C after the addition of iNOS assay reaction solution (100 μL, 50% NOS assay buffer, 39.8% MilliQ water, 5% l-Arginine solution, 5% 0.1 mM NADPH, and 0.2% DAF-FMDA) to each well. Parthenolide was used as a reference drug. A fluorescence plate reader was used to measure the fluorescences at excitation of 485 nm and emission of 528 nm [9]. The IC_50_ of each extract was determined.

**Inhibitory activity of methanolic extract (ME) against** **NO, NF‑кB, and TNF‑R2 production in LPS stimulated RAW 264.7 macrophages**

NO was determined in terms of its end product, nitrite, using Griess reagent after 24 hours of incubation with LPS [10]. Each cell lysate (100 µL) was mixed with 100 µL Griess reagent (1% sulfanilamide, 0.1% naphthylethylenediamine dihydrochloride, and 5% phosphoric acid), then incubated at room temperature for 10 min. The absorbances were recorded at 540 nm. Parthenolide was used as a positive control. The inhibition percentage was calculated using the following formula:

% Inhibition= [(OD control−OD sample)/ OD control x 100]. Where OD: optical denidensity concentrations at which the tested samples produced 50% inhibition of NO (IC_50_) were determined.

NF-*K*B, and TNF-R2 were determined by ELISA kit (#79756, BPS Bioscience, USA).

**Effect of methanolic extract (ME) on cytokines secretion (TNF-α, IL-1β, and IL-6) in LPS stimulated RAW 264.7 macrophages**

The released cytokines [TNF-*α* (ab181421), IL-1*β* (ab46052), IL-6 (ab178013)] were measured in the cell lysates using their respective ELISA kit according to the manufacturer’s instructions (abcam®, Human ELISA kit; China).

**Statistical analysis**

All analyses were performed in triplicate. Data were analyzed using GraphPad Prism 8® (San Diego, CA, USA) and represented as means (n=3) ± SD. Sample t-test and One-way ANOVA were carried out to determine significant differences among means followed by Tukey’s multiple comparison test.

**HPLC-DAD quantification of major identified phenolic compounds in the methanolic extract of *T. tipu* flowers**

HPLC system was performed on Waters Alliance 2695e with binary HPLC pump equipped with Waters 2996 PDA detector. Empower 2 software was used for both data collection and processing. A C18 column (150mm × 4.6mm I.D., 5μm) was used for the analysis. The column temperature was retained at 25±1^o^C. A ternary linear elution gradient [(A) water 0.2 % H3PO4 (v/v), (B) methanol, and (C) acetonitrile] with a flow rate of 1mL/min was used. Analysis was done on three biological replicates. While peak area measurement allowed for quantitative determination, the retention times of the peaks were compared with those of the reference phenolics to provide a quantitative determination.

**Computational analysis**

**System preparation and molecular docking**

The crystal structures of human cyclooxygenase-2, human 5-lipoxygenase, and human nitric oxide synthase (NOS) synthesize enzymes were obtained from the protein data bank and created using UCSF Chimaera [11]. Using PROPKA, the pH was fixed and optimized at 7.5 [12]. Drawings of the derived 2D structures were produced using ChemBioDraw Ultra 12.1 [13]. The Avogadro program [14] was used to optimize the 2D structure for energy reduction using the steepest descent method and MMFF94 force field. In order to prepare for docking with UCSF Chimaera, hydrogen atoms were removed [11].

**Molecular dynamic (MD) simulations**

The GPU-based PMEMD engine from the AMBER 18 package was used for all systems' MD simulations [15]. The partial atomic charge of each compound was ascertained using the General Amber Force Field (GAFF) approach from ANTECHAMBER [16].

The Leap module of the AMBER 18 package implicitly solvated each system inside an orthorhombic box of TIP3P water molecules within 10 of any box edge. Na+ and Cl- counter ions were added to each solution to bring it to equilibrium.

Each system experienced a 1000-step full minimization using the conjugate gradient algorithm without constraints after undergoing a 2000-step initial minimization with a 500 kcal/mol applied restraint potential.

Each system was gradually heated from 0K to 300K during a 500ps interval in order to guarantee that all systems had the same number of atoms and volume during the MD simulation. The solutes in the system were constrained by a 10 kcal/mol potential harmonic potential and a collision frequency of 1 ps. Additionally, 500ps of heating and equilibration at a constant temperature of 300K were performed on each system. In order to represent an isobaric-isothermal (NPT) ensemble, the number of atoms and system pressure were held constant for each production simulation, with the system's pressure being maintained at 1 bar using the Berendsen barostat [17].

For 20 ns, each system underwent MD simulation. The SHAKE approach was used to restrict the hydrogen bond atoms in each simulation. Each simulation has a 2fs step size and an SPFP precision model. The simulations were run with a random seeded isobaric-isothermal ensemble (NPT), a Langevin thermostat, a constant pressure of 1 bar, a pressure-coupling constant of 2ps, and a temperature of 300K.

**Post-MD Analysis**

After the trajectories had been saved using MD simulations at intervals of 1 ps, they were analyzed using the CPPTRAJ (Roe and Cheatham III, 2013) module of the AMBER18 suite. All graphs and visuals were produced with the Origin [18] data and Chimera [11] data analysis programs.

**Thermodynamic calculation**

The Poisson-Boltzmann or generalized Born and surface area continuum solvation (MM/PBSA and MM/GBSA) approach is useful for estimating ligand-binding affinities [19-21]. The exact statistical-mechanical binding free energies are calculated by the MM/GBSA and MM/PBSA Protein-Ligand complex molecular simulations within a predefined force field.

Over 200 snapshots taken over the whole 20 ns trajectory were used to average the binding free energy. For each molecular species (complex, ligand, and receptor), the estimation of the change in binding free energy (ΔG) can be shown as follows [22] :

$$\Delta G_{\mathrm{bind}}=G_{\mathrm{complex}}-G_{\mathrm{receptor}}-G_{\mathrm{ligand}} \left( 1 \right)$$

$$\Delta G_{\mathrm{bind}}=E_{\mathrm{gas}}+G_{\mathrm{sol}}-TS \left( 2 \right)$$

$$E_{\mathrm{gas}}=E_{\mathrm{int}}+E_{\mathrm{vdw}}+E_{\mathrm{ele}} \left( 3 \right)$$

$$G_{\mathrm{sol}}=G_{\mathrm{GB}}+G_{\mathrm{SA}} \left( 4 \right)$$

$$G_{\mathrm{SA}}=\gamma SASA \left( 5 \right)$$

The terms Egas, Eint, Eele, and Evdw stand for internal energy, van der Waals energy, Coulomb energy, and gas-phase energy, respectively. The Egas was calculated directly using the FF14SB force field terms. Calculating the solvation-free energy (Gsol) utilized the energy from the polar states (GGB) and non-polar states (G). The non-polar solvation free energy (GSA) was calculated from the Solvent Accessible Surface Area (SASA) using a water probe radius of 1.4 [23, 24].

In contrast, the polar solvation (GGB) contribution was evaluated by solving the GB equation. Items S and T stand for the total entropy of the solute and temperature, respectively. The contribution of each residue to the total binding free energy was calculated by the MM/GBSA-binding free energy method in Amber18.

**Supplementary figures**

|  |
| --- |
| **Fig. S1** Line graph representing the effect of the methanolic extract of *T. tipu* flowers on the RAW264.7 macrophages viability using the MTT assay. |


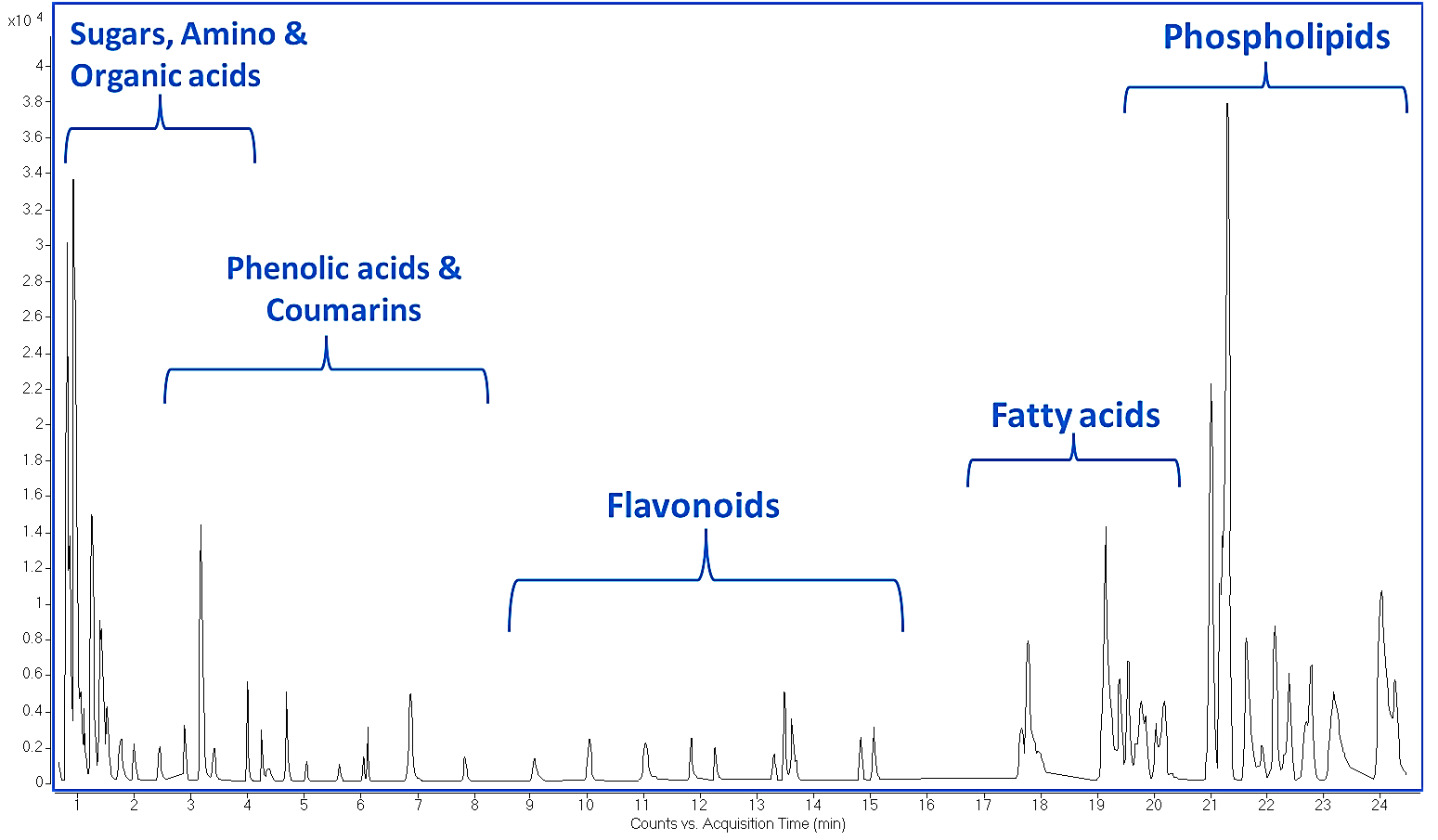


**Fig. S2.** LC-MS chromatogram of the methanolic extract of *T. tipu* flowers obtained in negative ion mode.


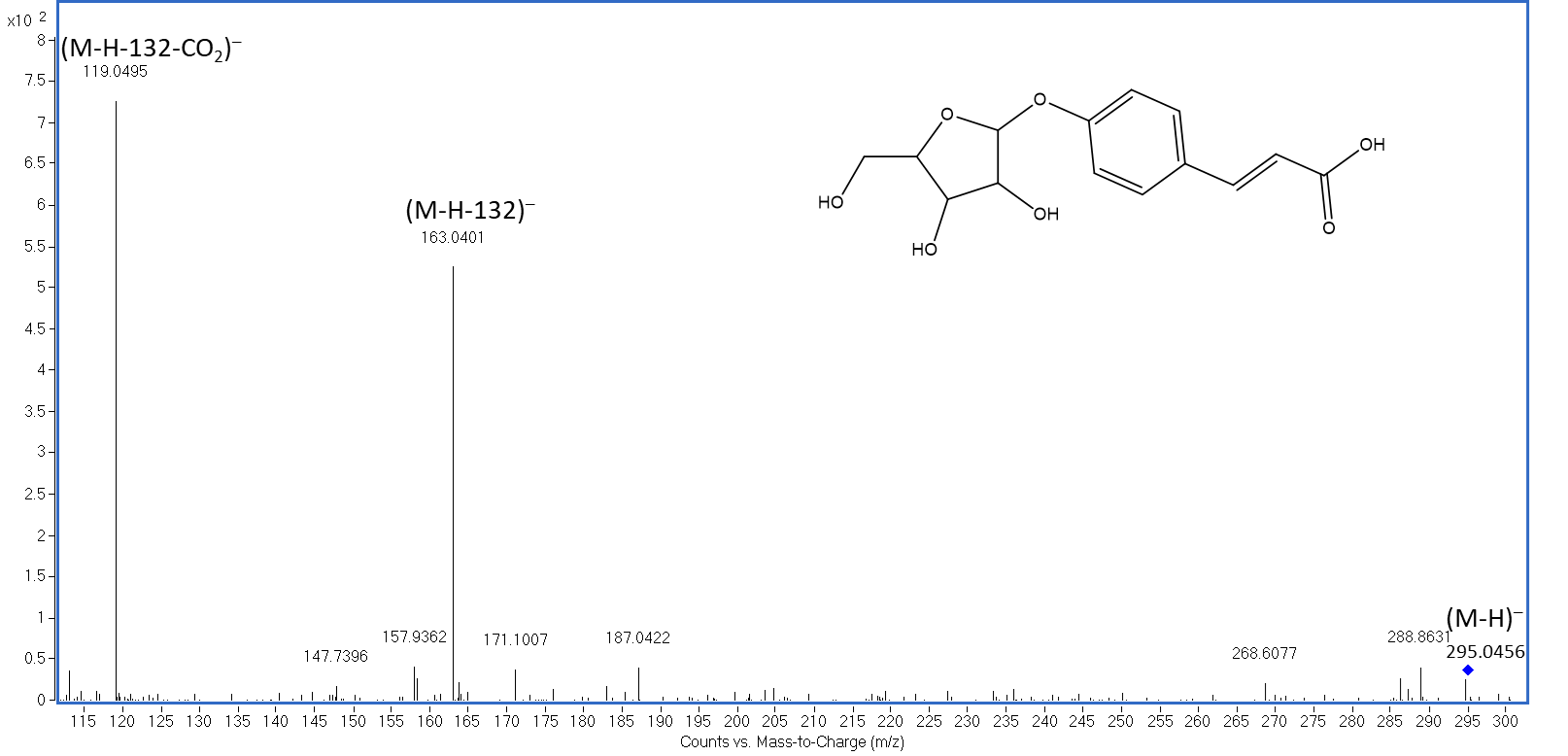


**Fig. S3.** MS/MS spectra obtained for coumaric acid pentoside (Compound 23, Table 1)

**
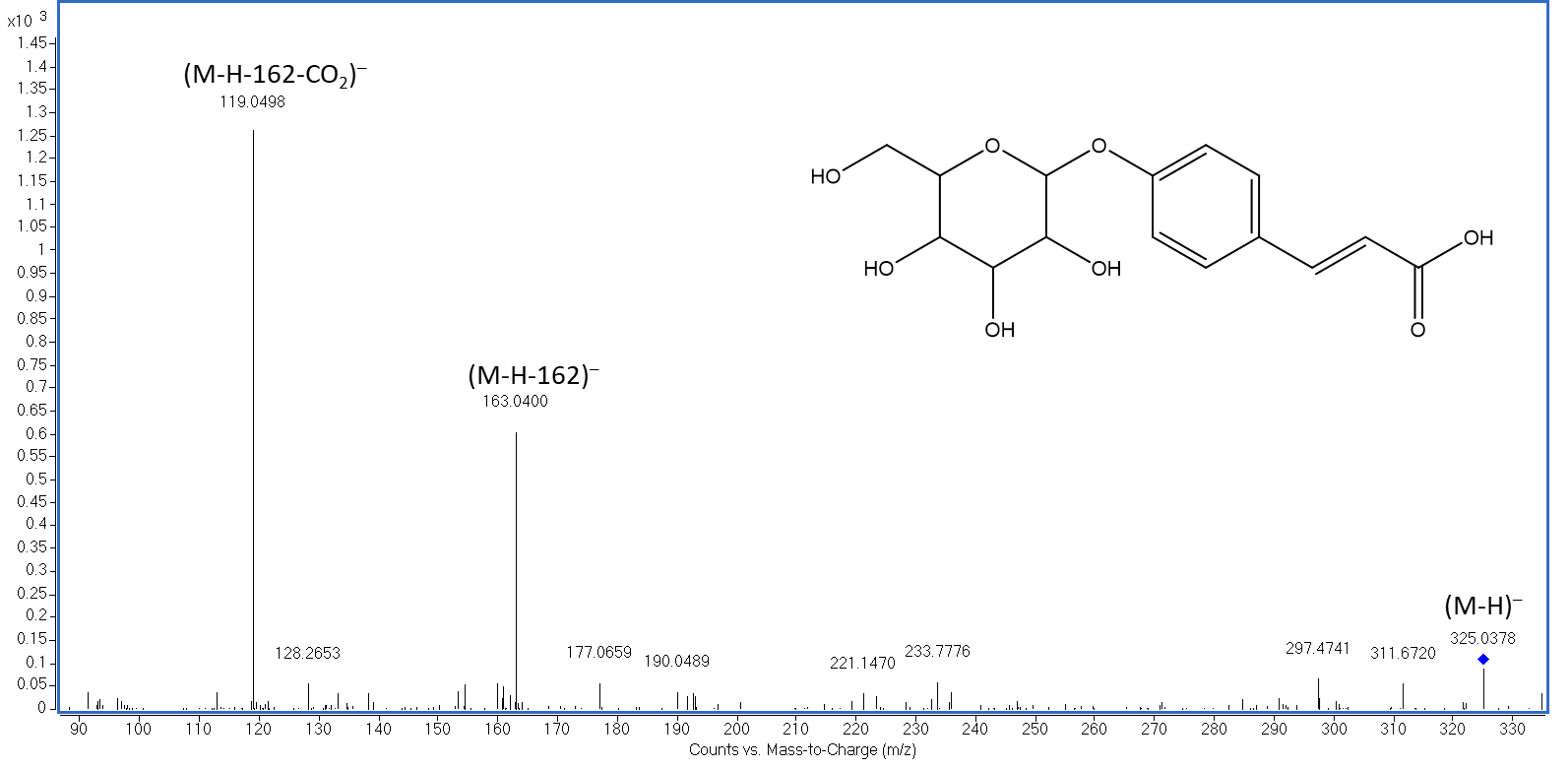
Fig. S4.** MS/MS spectra obtained for coumaric acid glucoside (Compound 24, Table 1)


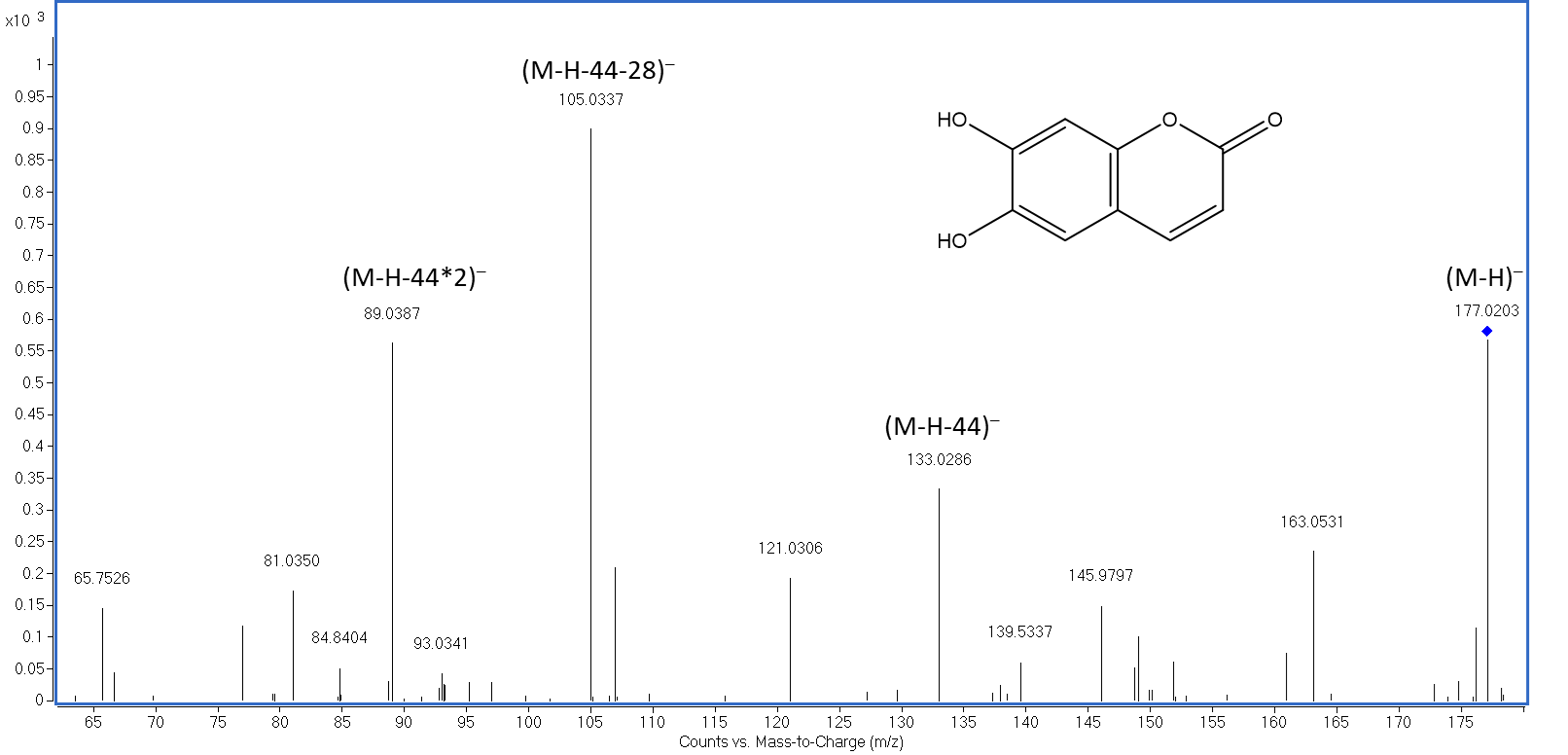


**Fig. S5.** MS/MS spectra obtained for esculetin (Compound 30, Table 1)

**
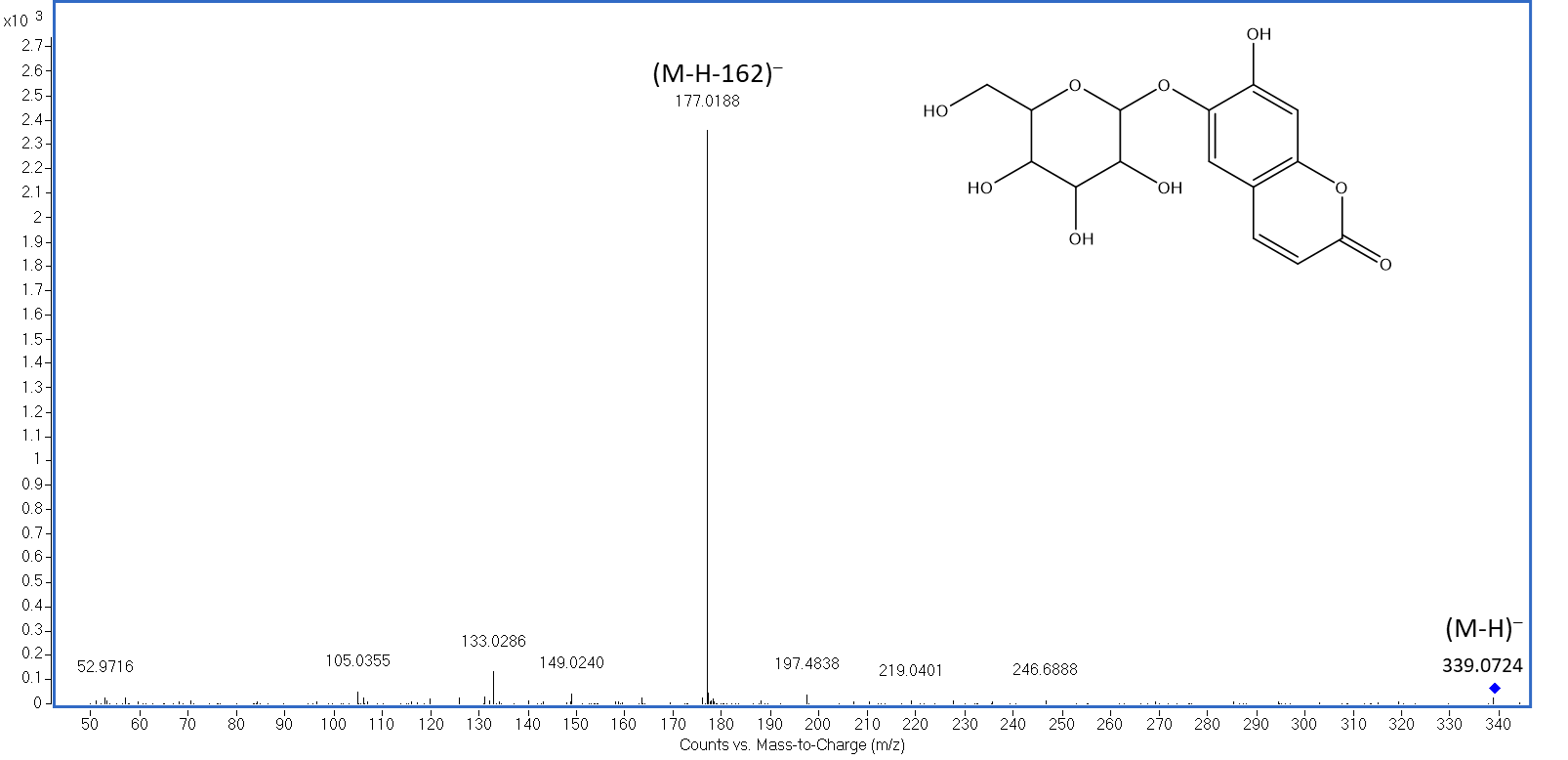
**

**Fig. S6.** MS/MS spectra obtained for esculin (Compound 29, Table 1)

**
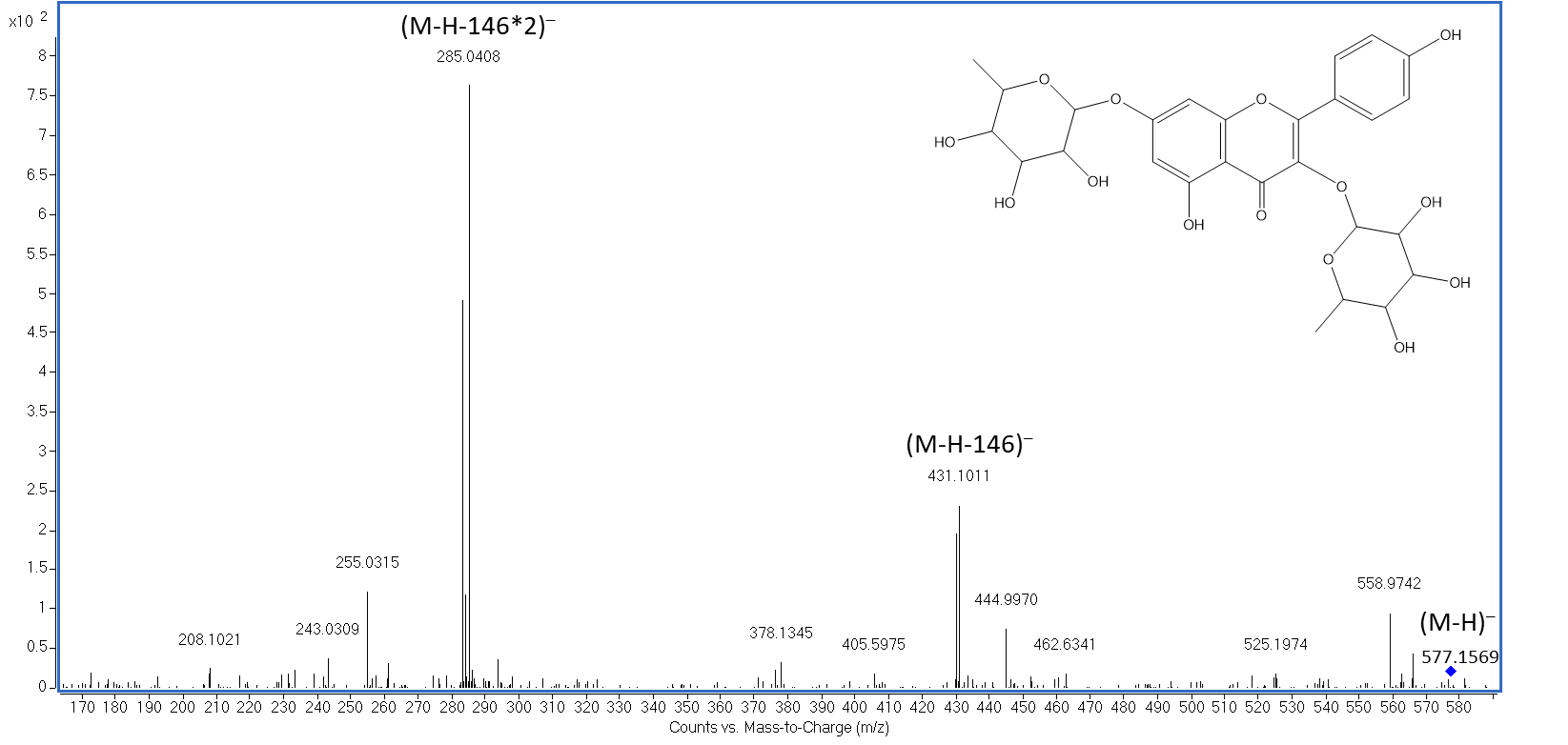
Fig. S7.** MS/MS spectra obtained for kaempferitrin (Compound 35, Table 1)

**
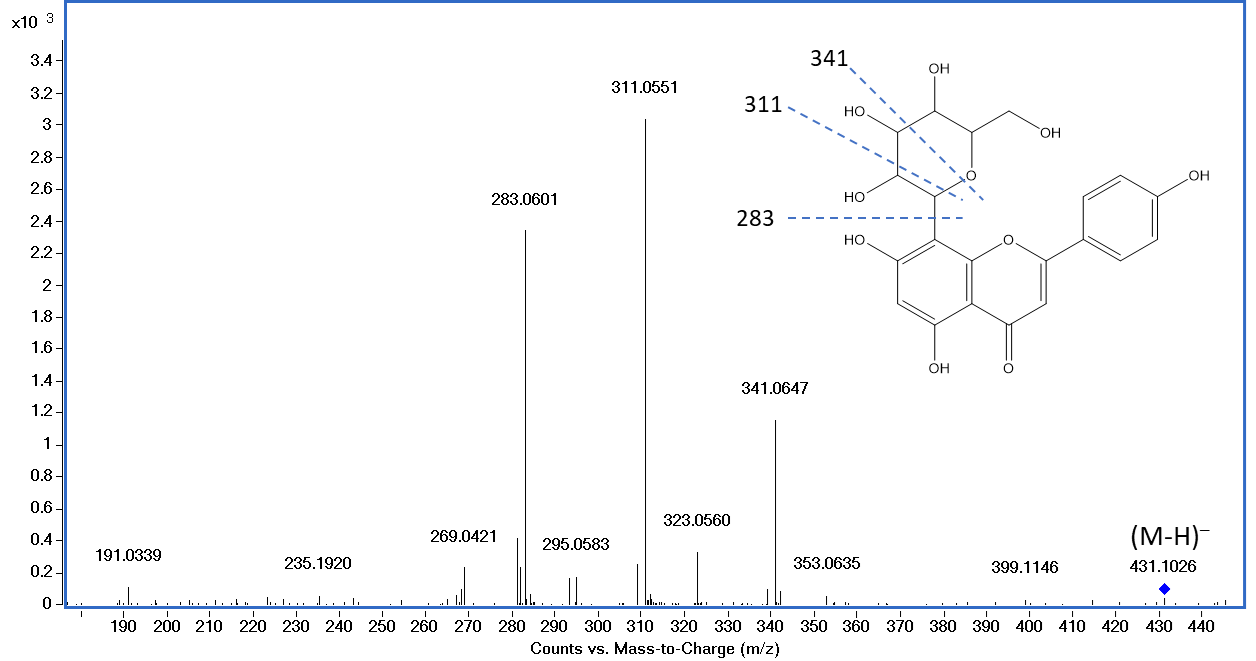
**

**Fig. S8.** MS/MS spectra obtained for vitexin (Compound 40, Table 1)

**
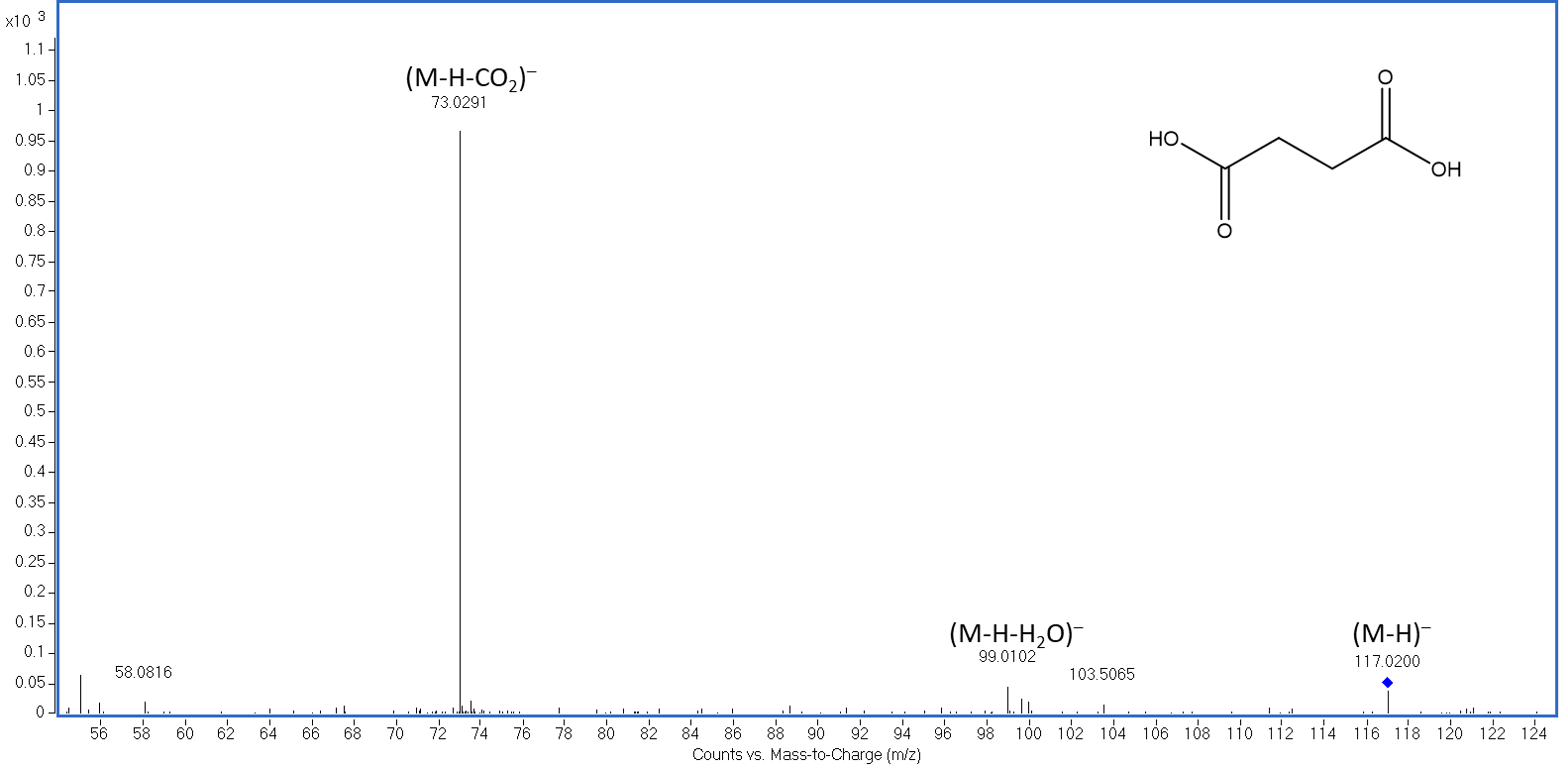
**

**Fig. S9.** MS/MS spectra obtained for succinic acid (Compound 40, Table 1)

**
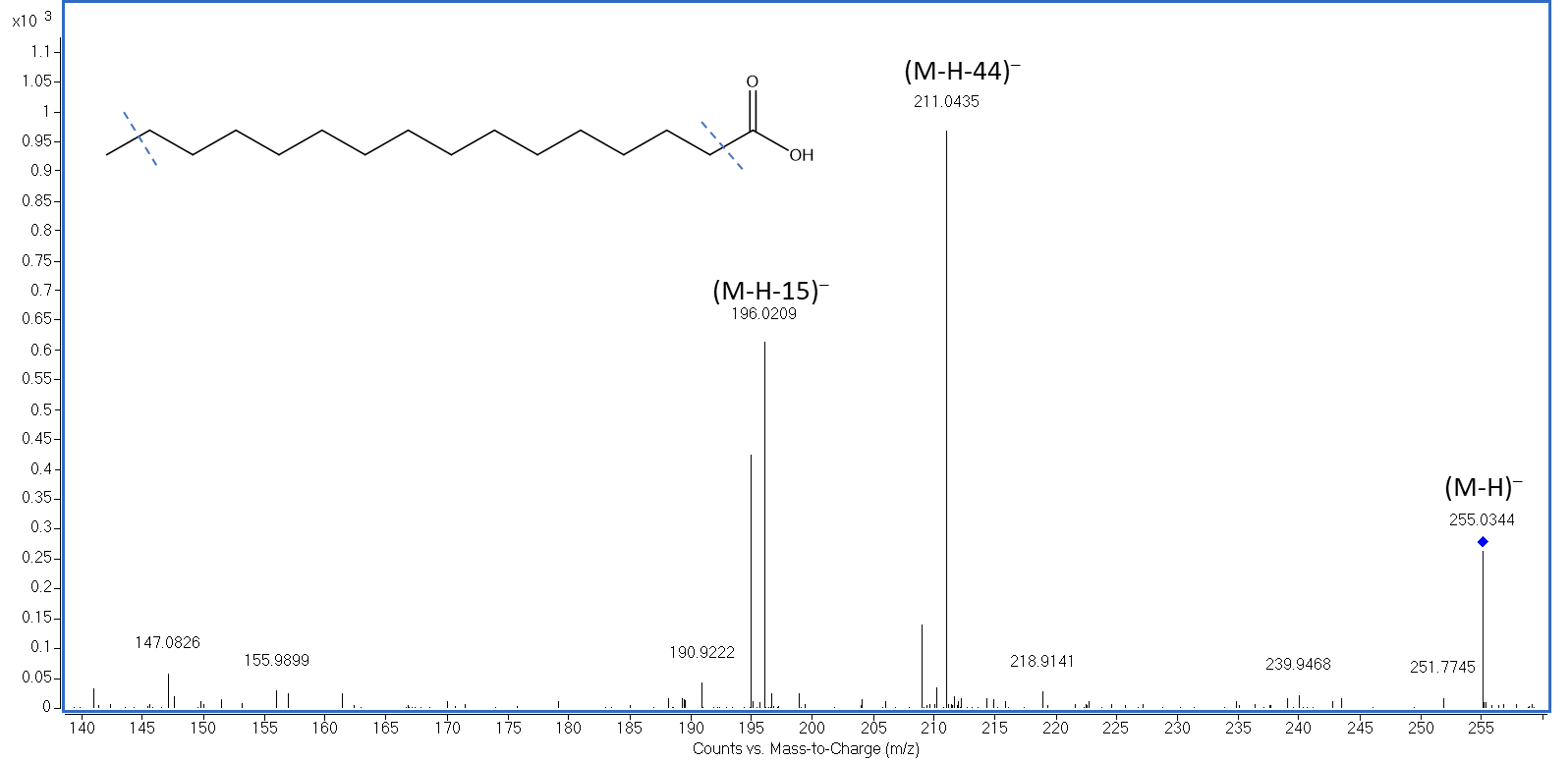
Fig. S10.** MS/MS spectra obtained for palmitic acid (Compound 51, Table 1)


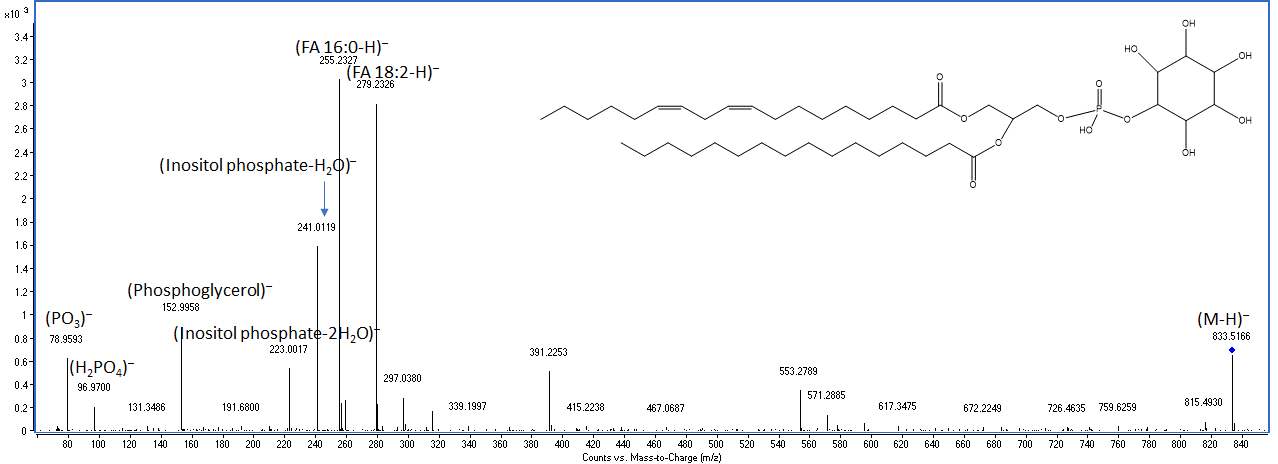
**Fig. S11.** MS/MS spectra obtained for PI(18:2/16:0) (Compound 61, Table 1)


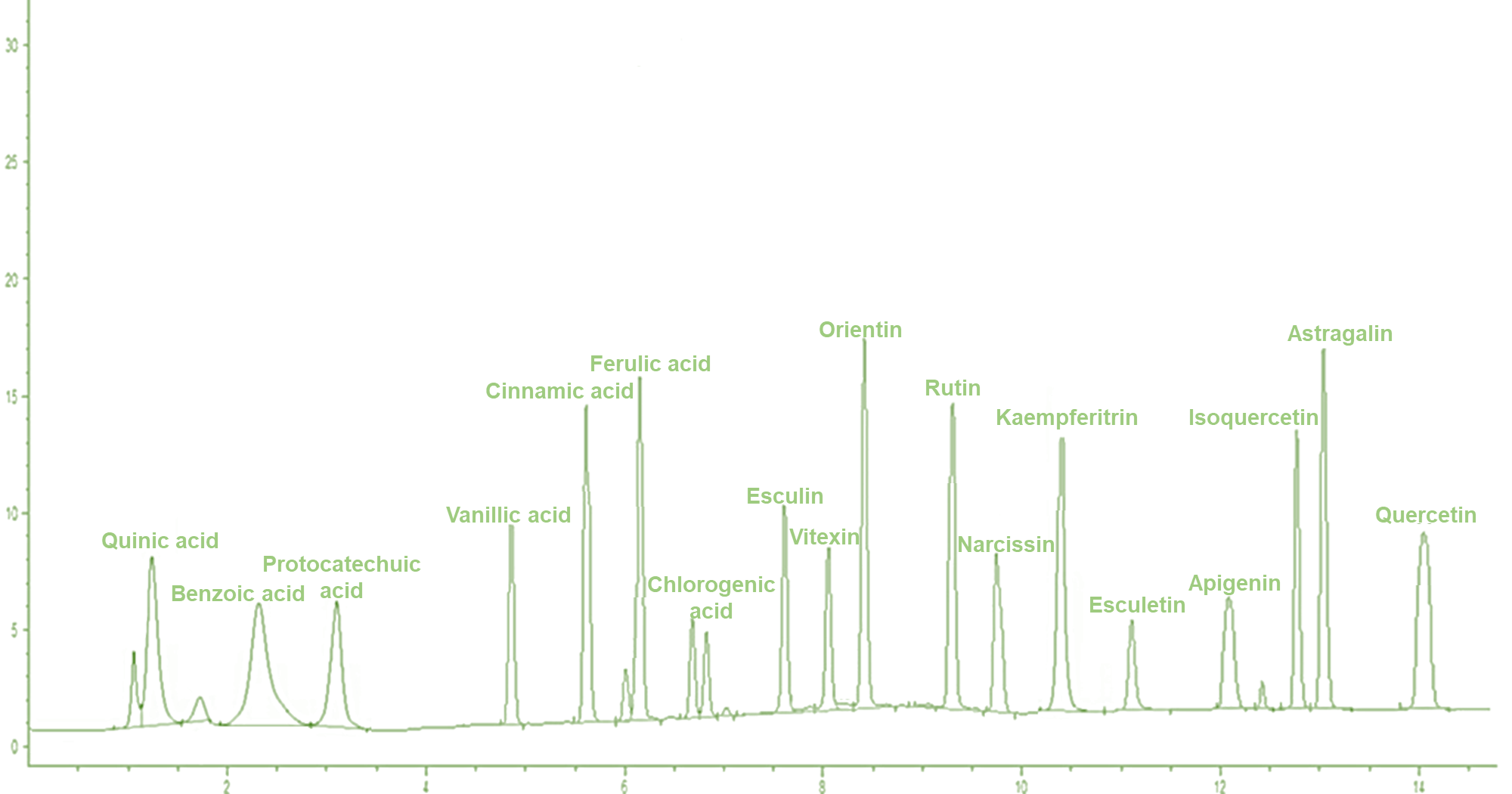


**Fig. S12.** HPLC-DAD chromatogram for quantification of major identified phenolic compounds in the methanolic extract of *T. tipu* flowers.

**References**

1. Abdel-Baki PM, Ibrahim RM, Mahdy NE. *Ferocactus herrerae* fruits: nutritional significance, phytochemical profiling, and biological potentials. Plant Foods Hum Nutr. 2022; 77: 545-551. DOI: https://doi.org/10.1007/s11130-022-01007-9

2. Martins N, Barros L, Duenas M et al. Characterization of phenolic compounds and antioxidant properties of *Glycyrrhiza glabra* L. rhizomes and roots. RSC Adv. 2015; 5: 26991-26997. DOI: https://doi.org/10.1039/C5RA03963K

3. Faitanin RD, Gomes JV, Rodrigues PM et al. Chemical study and evaluation of antioxidant activity and α-glucosidase inhibition of Myrciaria strigipes O. Berg (Myrtaceae). Journal of Applied Pharmaceutical Science. 2018; 8: 120-125. DOI: https://doi.org/10.7324/JAPS.2018.8317

4. Justino AB, Miranda NC, Franco RR et al. Annona muricata Linn. leaf as a source of antioxidant compounds with in vitro antidiabetic and inhibitory potential against α-amylase, α-glucosidase, lipase, non-enzymatic glycation and lipid peroxidation. Biomedicine & Pharmacotherapy. 2018; 100: 83-92. DOI: https://doi.org/10.1016/j.biopha.2018.01.172

5. George A, Chinnappan S, Chintamaneni M et al. Anti-inflammatory effects of Polygonum minus (Huds) extract (Lineminus™) in in-vitro enzyme assays and carrageenan induced paw edema. BMC Complement Altern Med. 2014; 14: 1-7. DOI: https://doi.org/10.1186/1472-6882-14-355

6. Costamagna MS, Zampini IC, Alberto MR et al. Polyphenols rich fraction from Geoffroea decorticans fruits flour affects key enzymes involved in metabolic syndrome, oxidative stress and inflammatory process. Food Chem. 2016; 190: 392-402. DOI: https://doi.org/10.1016/j.foodchem.2015.05.068

7. Manosroi A, Saraphanchotiwitthaya A, Manosroi J. Immunomodulatory activities of Clausena excavata Burm. f. wood extracts. J Ethnopharmacol. 2003; 89: 155-160. DOI: https://doi.org/10.1016/s0378-8741(03)00278-2

8. Jeong HY, Sung G-H, Kim JH et al. Syk and Src are major pharmacological targets of a Cerbera manghas methanol extract with kaempferol-based anti-inflammatory activity. J Ethnopharmacol. 2014; 151: 960-969. DOI: https://doi.org/10.1016/j.jep.2013.12.009

9. Zhao F, Wang L, Liu K. *In vitro* anti-inflammatory effects of arctigenin, a lignan from *Arctium lappa* L., through inhibition on iNOS pathway. J Ethnopharmacol. 2009; 122: 457-462. DOI: https://doi.org/10.1016/j.jep.2009.01.038

10. Picerno P, Autore G, Marzocco S et al. Anti-inflammatory activity of verminoside from kigelia a fricana and evaluation of cutaneous irritation in cell cultures and reconstituted human epidermis. J Nat Prod. 2005; 68: 1610-1614.

11. Pettersen EF, Goddard TD, Huang CC et al. UCSF Chimera—a visualization system for exploratory research and analysis. J Comput Chem. 2004; 25: 1605-1612. DOI: https://doi.org/10.1002/jcc.20084

12. Li H, Robertson AD, Jensen JH. Very fast empirical prediction and rationalization of protein pKa values. Proteins. 2005; 61: 704-721. DOI: https://doi.org/10.1002/prot.20660

13. Halford B. Reflections on CHEMDRAW. Chem Eng News. 2014; 92: 26-27.

14. Hanwell MD, Curtis DE, Lonie DC et al. Avogadro: an advanced semantic chemical editor, visualization, and analysis platform. J Cheminformatics. 2012; 4: 1-17. DOI: https://doi.org/10.1186/1758-2946-4-17

15. Lee T-S, Cerutti DS, Mermelstein D et al. GPU-accelerated molecular dynamics and free energy methods in Amber18: performance enhancements and new features. J Chem Inf Model. 2018; 58: 2043-2050. DOI: https://doi.org/10.1021/acs.jcim.8b00462

16. Wang J, Wang W, Kollman PA et al. Automatic atom type and bond type perception in molecular mechanical calculations. J Mol Graph Model. 2006; 25: 247-260. DOI: https://doi.org/10.1016/j.jmgm.2005.12.005

17. Berendsen HJ, Postma Jv, Van Gunsteren WF et al. Molecular dynamics with coupling to an external bath. J Chem Phys. 1984; 81: 3684-3690. DOI: https://doi.org/10.1063/1.448118

18. Seifert E. OriginPro 9.1: scientific data analysis and graphing software-software review. J Chem Inf Model. 2014; 54: 1552.

19. Kollman PA, Massova I, Reyes C et al. Calculating structures and free energies of complex molecules: combining molecular mechanics and continuum models. Acc Chem Res. 2000; 33: 889-897. DOI: https://doi.org/10.1021/ar000033j

20. Hayes JM, Archontis G. MM-GB (PB) SA calculations of protein-ligand binding free energies. Molecular dynamics-studies of synthetic and biological macromolecules. 2012; 171-190.

21. Ylilauri M, Pentikäinen OT. MMGBSA as a tool to understand the binding affinities of filamin–peptide interactions. J Chem Inf Model. 2013; 53: 2626-2633. DOI: https://doi.org/10.1021/ci4002475

22. Hou T, Wang J, Li Y et al. Assessing the performance of the MM/PBSA and MM/GBSA methods. 1. The accuracy of binding free energy calculations based on molecular dynamics simulations. J Chem Inf Model. 2011; 51: 69-82. DOI: https://doi.org/10.1021/ci100275a

23. Sitkoff D, Sharp KA, Honig B. Accurate calculation of hydration free energies using macroscopic solvent models. J Phys Chem. 1994; 98: 1978-1988. DOI: https://doi.org/10.1021/j100058a043

24. Greenidge PA, Kramer C, Mozziconacci J-C et al. MM/GBSA binding energy prediction on the PDBbind data set: successes, failures, and directions for further improvement. J Chem Inf Model. 2013; 53: 201-209. DOI: https://doi.org/10.1021/ci300425v
